# Patchy Phylogenetic Distribution and Poor Translational Adaptation of a Nested ORF in the Mammalian Mitochondrial *cytb* Gene

**DOI:** 10.3390/genes16070833

**Published:** 2025-07-17

**Authors:** Sheng-Lin Shi, Dan-Tong Li, Yan-Qun Liu

**Affiliations:** College of Bioscience and Biotechnology, Shenyang Agricultural University, Shenyang 110866, China; shishenglin@syau.edu.cn (S.-L.S.);

**Keywords:** *cytb* gene, nested ORF, mitochondrial protein, codon adaptation index, codon usage similarity, dispensable gene, Kozak sequence

## Abstract

Background: The mammalian mitochondrial genome has long been considered to encode only 13 proteins. However, a recent study identified a nested alternative open reading frame (nAltORF) within the primate mitochondrial *cytb* gene, which we designate *ncytb*, that is reportedly translated in the cytosol using the standard genetic code. This discovery challenges conventional understanding and raises questions about the prevalence, conservation, and translational adaptation of such ORFs. Methods: This study conducted a comprehensive bioinformatic analysis of nested *ncytb* genes in 289 primate and 380 rodent mitochondrial *cytb* sequences. Results: Nested *ncytb* genes meeting the criteria (>150 codons, standard genetic code) were identified in only 10.73% of primate and 20.53% of rodent species, suggesting a patchy phylogenetic distribution. While their encoded proteins showed homology to the previously reported protein encoded by the *Homo sapiens* nested *ncytb* gene, overall amino acid conservation was low, and characteristic protein domains or signal peptides were generally not predicted. Crucially, the Kozak consensus sequences surrounding the putative start codons of these *ncytb* genes were exclusively “weak” or “adequate”, with none classified as “strong” or “optimal”. Codon Adaptation Index (CAI) and Relative Codon Deoptimization Index (RCDI) analyses of the nested *ncytb* genes revealed neither significant adaptation nor deoptimization to the codon usage of nuclear and mitochondrial genes. Furthermore, cosine similarity analysis indicated that *ncytb* genes exhibit significantly lower codon usage similarity to both nuclear and mitochondrial gene sets compared to their host *cytb* genes. Conclusions: These findings collectively suggest that while *ncytb* genes exist in some mammals, their inconsistent presence, weak translational initiation signals, and lack of adaptation to cytosolic codon usage characterize them as dispensable genetic elements rather than core functional genes.

## 1. Introduction

The “one gene-one protein” paradigm, while a foundational concept in molecular biology, has been increasingly nuanced by the discovery of complex transcriptional and translational mechanisms that expand the coding capacity of genomes. Among these are alternative open reading frames (AltORFs), particularly nested AltORFs (nAltORFs), which encode distinct proteins from within the primary coding sequence of another gene. These nAltORFs have been identified across a wide array of organisms, from viruses to eukaryotes, indicating a conserved evolutionary strategy to maximize genetic information density [1,2].

Mammalian cells employ two distinct genetic systems: cytosolic ribosomes utilize the standard genetic code (NCBI translation table 1), while mitochondrial ribosomes employ the vertebrate mitochondrial code (NCBI translation table 2). These codes differ significantly. For instance, in the standard code, AUG is the primary start codon, AGA and AGG encode arginine, and UGA is a stop codon. In contrast, the vertebrate mitochondrial code uses AUA as an additional start codon, treats AGA and AGG as stop codons, and translates UGA as tryptophan [3].

For decades, the mammalian mitochondrial genome was considered a compact and streamlined entity, encoding a canonical set of 13 proteins essential for oxidative phosphorylation, alongside tRNAs and rRNAs necessary for their translation within the organelle [4]. This established view was recently challenged by a 2024 study that discovered a functional, evolutionarily conserved nAltORF (designated *ncytb* in this study) embedded within the mitochondrial cytochrome b (*cytb*) gene in mammals [5]. Remarkably, this protein is reportedly translated in the cytosol using the standard genetic code, unlike its host *cytb* gene, which is translated within the mitochondria using the vertebrate mitochondrial genetic code, and subsequently imported into mitochondria to modulate early development [5]. This unconventional biogenesis pathway presents a significant biological conundrum [6].

The existing research on nAltORFs typically involves translation using the same genetic code and ribosomal machinery as the host ORF. The *cytb* gene represents a rare case where a single mRNA transcript, originating from a mitochondrial gene, must be interpreted by two different translational systems. The mRNA of the *Homo sapiens* mitochondrial *cytb* gene is translated by mitochondrial ribosomes within the organelle and, putatively, by cytoplasmic ribosomes in the cytosol [5]. However, the evidence for cytosolic translation of mtDNA-derived transcripts is not yet definitive, and several critical mechanistic questions remain unanswered. As emphasized by the author, future in-depth studies will be essential to elucidate the mechanisms underlying *cytb* mRNA export from mitochondria and subsequent cytoplasmic translation [5]. This duality imposes a unique evolutionary challenge. The efficiency of protein translation is heavily influenced by codon usage, which is adapted to the availability of specific tRNAs within a given cellular compartment [7]. Since nuclear and mitochondrial genes have evolved under different selective pressures, they exhibit distinct codon usage patterns. It is therefore unclear whether and how the *ncytb* ORF has adapted its codon usage for efficient expression in the cytosol.

This uncertainty raises critical questions: Is the *ncytb* gene a widespread and conserved feature across mammals, or an isolated evolutionary novelty? If present in other species, how conserved is its sequence and potential function? Most importantly, how does a gene nested within a mitochondrial protein-coding gene, presumably optimized for mitochondrial translation, adapt its codon usage to allow for efficient translation by the cytosolic machinery? A lack of adaptation would suggest inefficient protein production, potentially indicating that the nested gene is non-functional or of low biological significance.

Therefore, this study undertakes an extensive bioinformatic investigation of mitochondrial *cytb* genes from two major mammalian orders, Primates and Rodentia, to screen for nested *ncytb* genes. We aim to determine their prevalence, assess the conservation of their encoded amino acid sequences, predict key protein characteristics, and analyze their codon usage patterns. Specifically, we evaluate the strength of their putative translation initiation signals (Kozak sequences) and quantify their codon adaptation to the nuclear translational environment using metrics such as the Codon Adaptation Index (CAI), the Relative Codon Deoptimization Index (RCDI), and codon usage similarity. Our findings indicate that *ncytb* genes are not universally present, exhibit weak translational initiation signals, and show a lack of significant adaptation to cytosolic codon usage. These results lead us to propose that the *ncytb* gene is likely a dispensable genetic element rather than a core, universally essential gene.

## 2. Materials and Methods

### 2.1. Sequence Data Acquisition

A total of 290 non-redundant primate and 391 rodent mitochondrial *cytb* gene sequences were retrieved from the GenBank database on 10 October 2024. Sequences containing ambiguous bases (non-A, C, G, T) were removed to facilitate subsequent analysis. After removing one sequence from the primate dataset, 289 sequences remained, and after removing 11 sequences from the rodent dataset, 380 sequences remained (Table S1).

The codon usage tables of nuclear genes and mitochondrial genes corresponding to the species included in this study were downloaded from the Codon and Codon-Pair Usage Tables (CoCoPUTs) database (https://dnahive.fda.gov/dna.cgi?cmd=codon_usage&id=537&mode=cocoputs, last updated September 2021) [8]. Reference codon usage tables of nuclear genes were considered robust if derived from a minimum of 30 coding sequences (CDSs), where the average CDS length exceeded 100 codons. The codon usage data for primates were downloaded on 24 November 2024, and the data for rodents were downloaded on 4 December 2024.

### 2.2. Screening for Nested Open Reading Frames

The screening for nested ORFs within the mitochondrial *cytb* gene sequences was performed following the established criteria [5]: (1) translation using the standard genetic code (NCBI translation table 1); (2) scanning within the +2 and +3 reading frames; (3) a minimum length of 150 codons; and (4) selection of the longest nested ORF per reading frame when multiple candidates were present. A parallel ORF analysis using the mammal mitochondrial genetic code was also conducted. The ORF scan was performed locally using custom Perl scripts (available upon request) and the Sequence Manipulation Suite (SMS) program (v2.0) [9]. For clarity, any identified nested ORF within the mitochondrial *cytb* gene was designated as the *ncytb* gene in this study.

### 2.3. Homology Analysis of ncytb-Encoded Proteins

To confirm the homology of the identified ORFs, the translated amino acid sequences of the putative *ncytb* genes were aligned against the previously reported *H. sapiens* Ncytb protein using the NCBI protein-protein BLAST (BLASTp) tool (https://blast.ncbi.nlm.nih.gov/Blast.cgi, accessed on 12 November 2024). The alignment results were used to evaluate the reliability of the *ncytb* genes obtained in this study.

### 2.4. Multiple Sequence Alignment of ncytb-Encoded Proteins

Multiple sequence alignment of the predicted Ncytb protein sequences was performed using the ClustalW algorithm with default parameters, as implemented in MEGA (v11.0) [10]. The resulting alignment was visualized using GeneDoc (v2.7.0) [11]. The SMS program (v2.0) was used to calculate pairwise identity and similarity between the amino acid sequences [9].

### 2.5. Phylogenetic Tree of cytb-Encoded Proteins

The Cytb protein sequences were aligned using the ClustalW algorithm with default settings, as implemented in MEGA (v11.0) [10]. A maximum likelihood phylogenetic tree was then constructed using the IQ-TREE web server (accessed on 12 July 2025) [12], also with default parameters. The resulting tree was visualized using the iTOL web server (v7.2.1) (accessed on 13 July 2025) [13].

### 2.6. Analysis of Translational Context

To evaluate the potential for cytosolic translation, each full-length mitochondrial *cytb* transcript was first translated in silico using the standard genetic code to identify any premature termination codons (PTCs) that would disrupt the reading frame. This study also examined the base composition at positions −6 to −1 upstream and +4 downstream of the putative start codon (AUG) of each nested *ncytb* gene for Kozak motif analysis. Following established classifications [14], the translational initiation strength of the start codon was categorized as follows: (1) “Optimal:” GCCRCCAUGG; (2) “Strong:” NNNRNNAUGG; (3) “Adequate:” NNNRNNAUG(A/C/U) or NNN(C/U)NNAUGG; and (4) “Weak:” NNN(C/U)NNAUG(A/C/U).

### 2.7. Prediction of ncytb-Encoded Protein Properties

The following online services were employed to predict key features of the putative Ncytb proteins:

- MitoFates: https://mitf.cbrc.pj.aist.go.jp/MitoFates/cgi-bin/top.cgi, accessed on 4 December 2024, used with default parameters for mitochondrial presequence prediction [15];
- SignalP-6.0: https://services.healthtech.dtu.dk/services/SignalP-6.0/, accessed on 28 December 2024, used with the “Mode: slow” model for signal peptide prediction [16];
- DeepLoc-2.1: https://services.healthtech.dtu.dk/services/DeepLoc-2.1/, accessed on 28 December 2024, used with the “High-quality (Slow)” model for subcellular localization prediction [13];
- DeepTMHMM 1.0: https://services.healthtech.dtu.dk/services/DeepTMHMM-1.0/, accessed on 29 December 2024, used with default parameters for transmembrane region prediction [17];
- NCBI Conserved Domain Database (CDD): https://www.ncbi.nlm.nih.gov/Structure/cdd/wrpsb.cgi, accessed on 29 December 2024, searched with default parameters for functional domain identification [18];
- IPC 2.0: https://ipc2.mimuw.edu.pl/, accessed on 4 January 2025, used with default parameters for isoelectric point (pI) prediction [19].

### 2.8. Codon Adaptation Index (CAI)

The Codon Adaptation Index (CAI), which ranges from 0 to 1, quantifies the extent to which the codon usage of a gene resembles that of a highly expressed reference set [20]. The improved CAI provides a better implementation than the original CAI in predicting protein production [21]. In this study, we used the E-CAI server (http://genomes.urv.es/CAIcal/E-CAI, accessed on 9 December 2024) to calculate an improved CAI and its corresponding expected value (eCAI). The eCAI corrects the amino acid bias and base bias by generating 500 random sequences in the calculation and provides an upper one-sided confidence limit for the CAI [22]. The lower side confidence limit is calculated by subtracting the upper confidence limit from twice the eCAI values manually. When the CAI was within the confidence interval of the eCAI, the codon usage of the query sequence was considered statistically indistinguishable from random. If the CAI exceeds the upper or lower limit of the eCAI, the query sequence shows positive or negative adaptation to the reference sequence.

### 2.9. Relative Codon Deoptimization Index (RCDI)

The Relative Codon Deoptimization Index (RCDI) compares the codon usage of a query gene to a reference set of genes presumed to be under low translational selection pressure. An RCDI value of 1 indicates identical codon usage, while values greater than 1 suggest increasing deoptimization relative to the reference set [23]. The expected RCDI (eRCDI) is calculated from mock sequences with similar GC content and amino acid composition, serving as a null hypothesis to assess the significance of the observed RCDI [24]. We used the RCDI/eRCDI online server (http://genomes.urv.cat/CAIcal/RCDI, accessed on 8 December 2024) to calculate both indices [24]. The upper limit provided by the server and the lower limit calculated by us are used to indicate the significance of RCDI. If the RCDI exceeded the upper or lower limit of the eRCDI, the codon usage was considered significantly different from random expectation. If the RCDI fell within the range of the eRCDI, the codon usage was considered to be primarily determined by local GC content and amino acid composition.

### 2.10. Cosine Similarity Analysis

The similarity index *D*(A, B) of codon usage, calculated from the cosine similarity index *R*(A, B), is used to estimate codon adaptation and was unfortunately systematically misinterpreted—a high value was thought to indicate higher similarity, while the opposite is true [25]. In fact, the cosine similarity index (ranging from −1 to 1) provides a direct and robust method alone to measure the similarity of codon usage patterns between sets of genes [26]. In this investigation, we applied the cosine similarity index, calculated via Microsoft Excel (v2016), to compare the codon usage patterns of the host *cytb* genes and the nested *ncytb* genes against two reference sets: the organism’s nuclear genes and its mitochondrial genes.

## 3. Results

### 3.1. Patchy Phylogenetic Distribution of the Nested ncytb Gene

Only the scan using the standard genetic code yielded positive results, while the scan using the mitochondrial genetic code yielded no results. Screening of mitochondrial *cytb* gene sequences from 289 primate and 380 rodent species revealed a sporadic distribution of the nested *ncytb* gene. In primates, 31 sequences (10.73%) possessed an *ncytb* gene that met the screening criteria. A higher prevalence was observed in rodents, where 78 sequences (20.53%) were found to be *ncytb*-positive. In all identified cases for both orders, the *ncytb* gene was located in the +3 reading frame, with no qualifying ORFs found in the +2 frame (Table S1). Several identical *ncytb* sequences were identified and consolidated to avoid redundancy. After removing duplicates, a set of 28 unique primate and 77 unique rodent *ncytb* genes was used for subsequent analyses.

**Figure 1:**
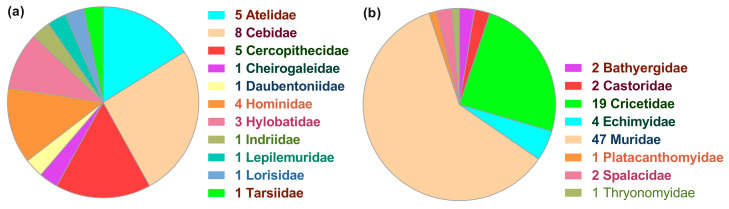
Taxonomic distribution of species with a nested ORF (*ncytb*) in their mitochondrial *cytb* gene. (**a**) Primates, (**b**) Rodentia. The number preceding the family name indicates the number of species analyzed within that family.

The taxonomic distribution of *ncytb*-positive species was inconsistent across families and genera. The 31 primate species that harbor *ncytb* genes were distributed across 16 genera and 11 families, with the number of representative species per family ranging from one to eight (Figure 1a). The 78 rodent species that harbor *ncytb* genes spanned a wider range of 35 genera within eight families (1–47 species per family) (Figure 1b).

**Figure 2:**
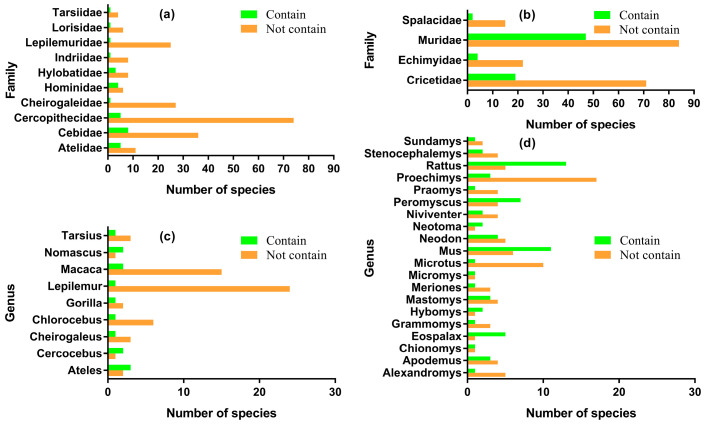
Comparison of the number of species with and without the nested *ncytb* gene at the family and genus taxonomic levels. (**a**) Family level in Primates, (**b**) Family level in Rodentia, (**c**) Genus level in Primates, (**d**) Genus level in Rodentia.

Crucially, the presence of the *ncytb* gene was inconsistent even within the same taxonomic groups. In Primates, the 258 species lacking the *ncytb* gene were distributed across 66 genera and 15 families. Notably, 10 of these families and nine of these genera contained both *ncytb*-positive and *ncytb*-negative species, indicating significant intra-taxon variation (Figure 2a,c). A similar pattern was observed in Rodentia, where the 302 *ncytb*-negative species were found in 145 genera and 20 families. Four families and 20 genera contained species both with and without the *ncytb* gene (Figure 2b,d). In the phylogenetic trees of Cytb proteins for both Primates (Figure S1) and Rodentia (Figure S2), most clades contain a mix of both *ncytb*-positive species (marked in green) and *ncytb*-negative species (marked in red). Only a few small clades are composed exclusively of *ncytb*-positive species.

### 3.2. Sequence Homology of Putative ncytb-Encoded Proteins

To assess whether the identified ORFs were homologous to the functionally characterized *H. sapiens* Ncytb protein, the amino acid sequence of the *H. sapiens* Ncytb was used as a query for pairwise BLASTp analysis against all other putative Ncytb proteins (Table S2).

The analysis revealed significant sequence similarity across all comparisons. For the 27 other primate sequences, sequence identity ranged from 45.81% (*Tarsius bancanus*) to 94.12% (*H. sapiens neanderthalensis*), with sequence similarity (positives) ranging from 62.57% (*T. bancanus*) to 97.33% (*H. sapiens neanderthalensis*). All alignments were statistically significant (lowest bit score = 119; max E-value = 1.17 × 10^−37^). When compared to the 77 rodent Ncytb sequences, the *H. sapiens* protein showed identities between 40.00% (*Proechimys roberti*) and 55.77% (*Microtus kikuchii*), and similarities between 58.82% (*Thryonomys swinderianus*) and 71.27% (*Castor fiber*). These alignments were also highly significant (lowest bit score = 98.2; max E-value = 4.24 × 10^−29^).

### 3.3. Amino Acid Conservation of ncytb-Encoded Proteins

To evaluate the degree of conservation among the putative Ncytb proteins, multiple sequence alignments were performed separately for the primate and rodent datasets.

**Figure 3:**
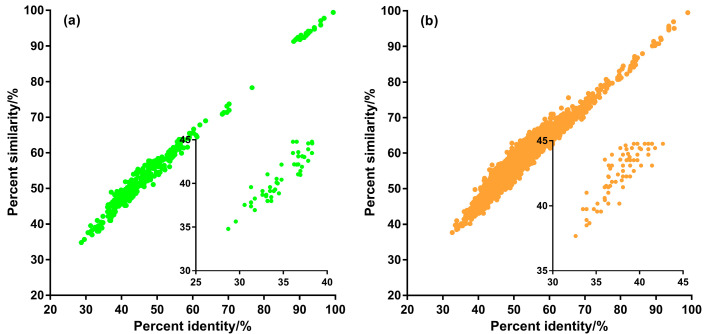
Scatter plot of pairwise percent identity versus percent similarity for full-length Ncytb protein sequences. (**a**) Primates (n = 351), (**b**) Rodentia (n = 2775).

Multiple sequence alignment of the 28 primate Ncytb protein sequences revealed one sequence (from NC_037853.1) that was a 152-amino-acid, 5′-truncated variant (Figure S3). This truncated sequence exhibited low identity (max 32.68%) and similarity (max 34.24%) with other primate sequences (Table S3). Across the full alignment of all 28 sequences, only three amino acid sites (L, R, Q) were invariant (Figure S3). After excluding the truncated variant, the alignment of the remaining 27 full-length sequences showed eight invariant sites (M, L, R, R, L, L, R, Q). Among these 27 full-length sequences, pairwise identity ranged from 28.70% to 99.42%, and similarity ranged from 34.78% to 99.42%. A quantitative analysis of all 351 pairwise comparisons showed that 72.65% of pairs had >40% identity, and 92.59% had >40% similarity (Table S3, Figure 3a).

A similar analysis of the 77 rodent Ncytb protein sequences identified two 5′-truncated variants (from NC_040138.1 and NC_003041.1), which were 156 and 173 amino acids long, respectively (Figure S4). In the alignment of all 77 sequences, only four amino acid sites (L, L, R, Q) were invariant. Excluding the two truncated sequences, there were 75 full-length proteins, in which 12 sites were invariant (M, L, R, L, R, L, L, S, L, L, R, Q). Among the 75 full-length rodent sequences, pairwise identity ranged from 32.64% to 98.93% and similarity ranged from 37.66% to 99.47%. Of the 2775 pairwise comparisons, 97.19% had >40% identity, and 99.68% had >40% similarity (Table S3, Figure 3b).

**Figure 4:**
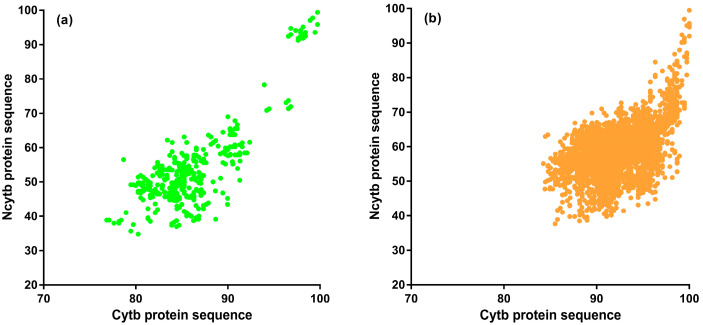
Pairwise sequence similarity comparison between Ncytb and Cytb protein sequences. (**a**) Primates (n = 351; Spearman’s *ρ* = 0.5986, *p* < 0.0001), (**b**) Rodentia (n = 2775; Spearman’s *ρ* = 0.4968, *p* < 0.0001).

Additionally, we investigated the evolutionary relationship between the Ncytb and Cytb proteins by examining pairwise sequence similarity correlations (Figure 4). The moderate, yet statistically significant, correlations observed in both Primates (Spearman’s *ρ* = 0.5986, *p* < 0.0001) and Rodentia (*ρ* = 0.4968, *p* < 0.0001) indicate distinct evolutionary trajectories for this protein pair. These findings imply that different selective pressures may be acting on the nested Ncytb protein compared to the mitochondrial Cytb protein, suggesting a divergence in their evolutionary paths.

### 3.4. Predicted Properties of ncytb-Encoded Proteins

The analysis of the predicted protein sequences revealed shared characteristics between primate and rodent Ncytb proteins, but a general lack of canonical functional features (Table S4). The 28 primate Ncytb proteins ranged in size from 152 to 230 amino acids, while the 77 rodent proteins ranged from 156 to 239 amino acids. Using MitoFates, a mitochondrial targeting presequence was predicted in only 9 of 28 primate and 52 of 77 rodent proteins. Notably, no presequence was predicted for the established *H. sapiens* Ncytb protein.

Furthermore, analysis with SignalP-6.0, DeepTMHMM, and the NCBI CDD server failed to identify any signal peptides, transmembrane regions, or conserved functional domains in any of the 105 primate and rodent Ncytb sequences. This lack of a predicted signal peptide for the *H. sapiens* Ncytb protein differs from a previous report [5]. Subcellular localization predictions by DeepLoc-2.1 suggested a diffuse distribution for the proteins, with the highest probabilities for the cytoplasm and extracellular space (Table S4).

Consistent with trends reported for other mammalian nested ORFs [2], the predicted Ncytb proteins were found to be highly basic. The isoelectric points (pI) of the 28 primate proteins ranged from 10.77 to 12.16 (mean = 11.61), and the 77 rodent proteins had pI values between 11.14 and 12.23 (mean = 11.70) (Table S4).

### 3.5. Cytosolic Integrity of the cytb Primary Open Reading Frame

The translation of *ncytb* in the cytosol is contingent on how cytosolic ribosomes interpret the host *cytb* mRNA. The UGA codon, which encodes tryptophan in mitochondria, is a termination codon in the standard genetic code. Therefore, we analyzed the *cytb* sequences for premature termination codons (PTCs) under the standard code.

Our analysis revealed that 104 of the 105 *cytb* genes contained an in-frame UGA codon at positions 88–90, with the remaining sequence containing it at positions 91–93 (Table S5). The presence of this UGA codon would cause translation of the primary *cytb* reading frame to terminate prematurely in the cytosol. A subsequent scan for alternative in-frame AUG codons downstream of this PTC failed to identify any that could initiate an ORF longer than 50 codons. This genomic architecture—an early termination of the primary ORF—leaves the downstream region containing the +3 reading frame of the *ncytb* gene structurally intact and potentially accessible to the translational machinery.

### 3.6. Context of the ncytb AUG Initiation Codon

We systematically analyzed the Kozak sequences surrounding the start codons of the 105 *ncytb* genes to classify their potential for translation initiation.

**Figure 5:**
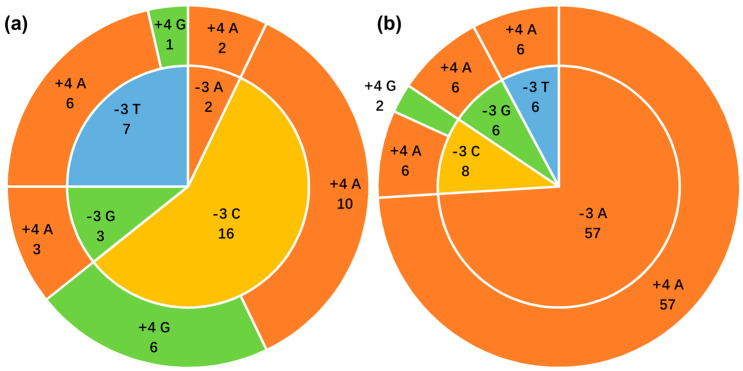
Nucleotide composition at the key −3 and +4 positions of the Kozak consensus sequence. (**a**) Primates, (**b**) Rodentia. The inner and outer pie charts depict the base composition at positions −3 and +4, respectively, relative to the AUG start codon. Numbers indicate the count of species with the specified base at that position.

Across both taxonomic orders, none of the 105 examined *ncytb* genes possessed a Kozak sequence classified as “optimal” or “strong.” An “adequate” context was identified in 12 of 28 primate genes and 65 of 77 rodent genes. The remaining sequences in both groups (16 in primates, 12 in rodents) displayed only a “weak” initiation context (Table S6, Figure 5).

### 3.7. Codon Adaptation and Deoptimization of the Nested ncytb Gene

Sufficiently deep codon usage data for the host nuclear genome was available for a subset of the identified species. Of the 28 primate *ncytb* genes, 12 had corresponding reference data, as did 28 of the 77 rodent *ncytb* genes (Table S4). Consequently, all subsequent codon usage analyses were restricted to this subset of 12 primate and 28 rodent genes.

**Figure 6:**
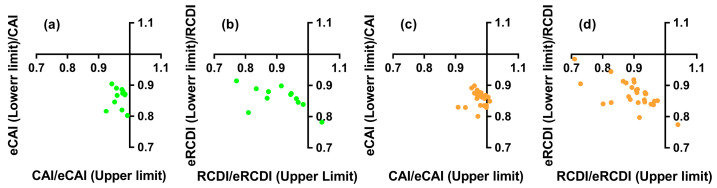
Codon adaptation of the nested ORF in the mitochondrial *cytb* gene to nuclear genes. (**a**) CAI analysis in Primates, (**b**) RCDI analysis in Primates, (**c**) CAI analysis in Rodentia, (**d**) RCDI analysis in Rodentia.

The analysis of the Codon Adaptation Index (CAI) and Relative Codon Deoptimization Index (RCDI) showed that the *ncytb* genes are not adapted to the codon usage of their host’s nuclear genes. For primates, all 12 CAI values fell within the 95% confidence interval of the expected CAI (eCAI), indicating no significant adaptation (Table S7, Figure 6a). Similarly, only one of the 12 RCDI values fell outside the expected range, suggesting a general lack of deoptimization (Table S7, Figure 6b). In rodents, only two of the 28 *ncytb* genes displayed CAI values outside the 95% confidence interval of the eCAI (Table S7, Figure 6c). The RCDI analysis yielded a consistent result, with only one of the 28 values exceeding the expected threshold (Table S7, Figure 6d).

### 3.8. Codon Usage Similarity of the Nested ncytb Gene

**Figure 7:**
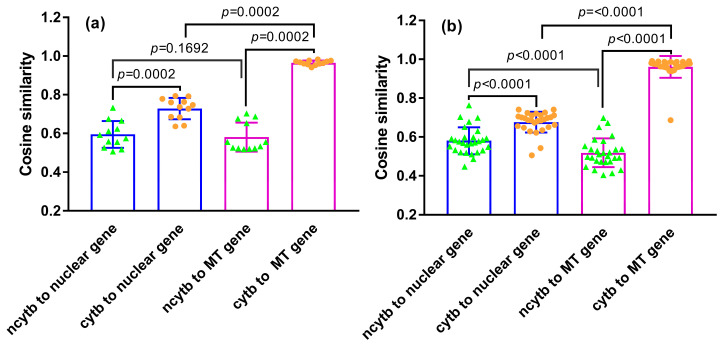
Codon usage similarity between the mitochondrial *cytb* gene and its nested ORF with nuclear and mitochondrial genes. (**a**) Primates, (**b**) Rodentia. Similarity comparisons were performed using the one-tailed Wilcoxon signed-rank test. Each comparison is visualized as a stacked plot combining a scatter plot and a bar chart. The color combinations indicate the pairwise comparison targets: green scatter plots over blue bars represent the codon usage similarity between the *ncytb* gene (green) and nuclear genes (blue); green scatter plots over purple bars represent the similarity between the *ncytb* gene and mitochondrial genes (purple). Similarly, orange scatter plots over blue bars represent the *cytb* gene (orange) compared with nuclear genes (blue), and orange scatter plots over purple bars represent the *cytb* gene compared with mitochondrial genes (purple).

To further investigate codon usage patterns, RSCU-based cosine similarity was used to compare the *ncytb* and host *cytb* genes against both nuclear and mitochondrial reference gene sets (Table S8, Figure 7).

In primates, the host *cytb* gene exhibited, as expected, significantly higher codon usage similarity to mitochondrial genes than to nuclear genes (Wilcoxon one-tailed test, *p* = 0.0002). In contrast, the nested *ncytb* gene showed significantly lower similarity to both nuclear and mitochondrial genes when compared to its host *cytb* gene (*p* = 0.0002 for both). No significant difference was found between the *ncytb* gene’s similarity to nuclear versus mitochondrial genes (*p* = 0.1692).

A similar pattern was observed in rodents. The *cytb* gene was again highly adapted to the mitochondrial environment (*p* < 0.0001), and the *ncytb* gene showed significantly lower similarity to both reference sets compared to its host (*p* < 0.0001 for both). However, unlike in primates, the rodent *ncytb* gene displayed a slightly but significantly higher codon usage similarity to nuclear genes than to mitochondrial genes (*p* < 0.0001).

## 4. Discussion

The central dogma of molecular biology has been progressively refined with the understanding that eukaryotic genomes are far more complex than initially perceived. Mechanisms like alternative splicing and the utilization of AltORFs significantly expand the proteomic landscape from a finite set of genes [27]. The discovery of functional nAltORFs, in particular, underscores that genetic information within eukaryotic mRNAs can be denser and more multifaceted than a simple one-to-one gene-protein correspondence [2,28]. Prompted by the recent identification of a functional nested ORF (the *ncytb* gene) within the human mitochondrial *cytb* gene, this study embarked on a comprehensive bioinformatic survey of homologous *ncytb* genes in Primates and Rodentia, yielding results that provide new insights into their nature and evolutionary trajectory.

A fundamental prerequisite for the expression of the *ncytb* gene in the cytosol is that the primary *cytb* reading frame must not interfere. Our integrity analysis of the *cytb* gene under standard genetic code conditions revealed a UGA stop codon near the 5′ end in nearly all sequences (Table S5). This premature stop codon would prevent the synthesis of full-length Cytb protein by cytosolic ribosomes. This finding is consistent with the proposed model where mitochondrial *cytb* mRNA, if transported to the cytoplasm, can serve as a template for *ncytb* synthesis without interference from the translation of the host gene [5].

A key finding of this research is the limited and patchy distribution of the *ncytb* gene. We detected *ncytb* genes meeting our criteria in only 10.73% of primate and 20.53% of rodent species analyzed. This sporadic presence, often varying even within the same genus or family (Figure 2, Figures S1 and S2), aligns with the characteristics of dispensable genes. Dispensable genes are defined as loci that can be absent in some individuals or lineages within a taxon without causing obvious deleterious phenotypic effects under standard conditions [29]. They are often associated with adaptive functions rather than core, housekeeping roles [30]. The inconsistent occurrence of the *ncytb* gene across the surveyed mammals strongly suggests it does not belong to the core mitochondrial or cellular proteome but rather fits the profile of a dispensable genetic element.

The amino acid sequences encoded by the identified *ncytb* genes exhibited homology to the experimentally verified *H. sapiens* Ncytb protein, as evidenced by BLASTp analysis (Table S2), suggesting a potential for conserved, albeit auxiliary, biological function [31,32]. However, multiple sequence alignments revealed a relatively low degree of overall amino acid conservation, with only a few invariant residues across primates and rodents (Figures S3 and S4). Furthermore, predictive analyses for signal peptides, transmembrane domains, or known functional domains did not yield positive identifications (Table S4). This lack of strong sequence conservation and identifiable domains, coupled with the high isoelectric points observed (Table S4)—a feature noted in other studies of nAltORF-encoded proteins—could imply that if these proteins are functional, their roles might be subtle or related to general properties like nucleic acid interaction rather than highly conserved enzymatic functions. This again points toward an auxiliary role, consistent with the concept of dispensability [33]. The discrepancy regarding the signal peptide prediction with the original report warrants further investigation; it could be due to sequence divergence leading to the loss of the feature in many species, or it may be a primate-specific or even human-lineage-specific feature [16].

A critical aspect for any gene to be functionally expressed is efficient translation initiation. Our analysis of the Kozak consensus sequence surrounding the putative AUG start codons of *ncytb* genes revealed a striking pattern: none possessed “optimal” or “strong” contexts. The majority were classified as “adequate” or “weak” (Figure 5, Table S6). Weak initiation contexts are known to result in inefficient translation, which implies that even if *ncytb* mRNA is available in the cytosol, its protein product may be synthesized at low levels [34].

The proposed dual-translation scenario for *cytb* mRNA—mitochondrial translation for Cytb protein and cytosolic translation for a “novel protein”—necessitates that the nested *ncytb* gene navigate the distinct codon usage environment of the cytosol. Our CAI and RCDI analyses (Figure 6, Table S7) demonstrated that the *ncytb* genes generally show no significant adaptation to the codon usage of their host species’ nuclear genes. Their CAI values largely fell within the range expected for random sequences, suggesting a lack of selective pressure to optimize codon usage for efficient cytosolic translation. This contrasts with highly expressed nuclear genes, which typically exhibit codon usage adapted to the cytoplasmic tRNA pool [35]. Similarly, the cosine similarity analysis (Figure 7, Table S8) showed that *ncytb* genes have significantly lower codon usage similarity to both nuclear and mitochondrial reference sets compared to the host *cytb* gene. The *cytb* gene exhibits significantly greater codon usage similarity to mitochondrial genes than to nuclear counterparts, whereas such preference is indistinguishable for the *ncytb* gene. The *ncytb* gene’s codon usage appears to be determined primarily by the sequence constraints of its host gene rather than being optimized for either cellular compartment. This lack of adaptation is a strong indicator of either very low expression levels, recent evolutionary origin, or a non-critical function that does not impose strong selective pressure for translational optimization [36]. Our findings diminish the likelihood of efficient cytosolic translation of the *cytb* gene and challenge both the general applicability and mechanistic stability of the mitochondrial mRNA export with protein return mechanism [6].

In conclusion, this study provides comprehensive bioinformatic evidence suggesting that the *ncytb* gene, while present in a subset of primate and rodent species, exhibits the characteristics of a dispensable gene. Its patchy phylogenetic distribution, low overall amino acid conservation, generally weak translation initiation signals, and, most notably, a lack of codon usage adaptation to the cytosolic translational machinery collectively argue against it being a core, essential gene. While it may perform auxiliary or species-specific functions in some mammals, its evolutionary trajectory appears distinct from the highly conserved mitochondrial genes. Future experimental work is needed to validate the expression and functional relevance of these *ncytb*-encoded proteins in the diverse species where they are found and to elucidate the mechanisms governing their potential translation in the cytosol. This research highlights the evolving understanding of mitochondrial genome complexity and the intriguing ways in which genetic information can be encoded and expressed.

## Data Availability

The datasets analyzed in this study were obtained from publicly available databases, including GenBank and codon usage databases. The original contributions and analyses presented in this article are included within the article. Further inquiries can be directed to the corresponding author.

## Supplementary Materials

The following supporting information can be downloaded at: https://www.mdpi.com/article/10.3390/genes16070833/s1, Figure S1: Phylogenetic tree of cytochrome b encoded proteins in Primates; Figure S2: Phylogenetic tree of cytochrome b encoded proteins in Rodentia; Figure S3: Multiple sequence alignment of cytochrome b nested gene-encoded proteins in Primates; Figure S4: Multiple sequence alignment of cytochrome b nested gene-encoded proteins in Rodentia; Table S1: Cytochrome b and its nested genes in Primates and Rodentia; Table S2: Pairwise alignment of the screened nested genes and the validated nested genes; Table S3: Amino acid sequence identity and similarity from multiple sequence alignment of nested genes in Primates and Rodentia; Table S4: Bioinformatics prediction of characteristics of proteins encoded by nested genes; Table S5: Detecting internal stop codons in cytochrome b genes under the standard genetic code across Primates and Rodentia; Table S6: Kozak sequences of genes nested within cytochrome b across Primates and Rodentia; Table S7: CAI and RCDI values of nested genes within cytochrome b in Primates and Rodentia; Table S8: Codon usage similarity between cytochrome b gene and its nested ORF with nuclear and mitochondrial genes.

## Abbreviations

The following abbreviations are used in this manuscript:

AltORF	alternative open reading frame
BLASTp	protein-protein BLAST
CAI	Codon Adaptation Index
CDS	coding sequences
CoCoPUTs	Codon and Codon-Pair Usage Tables
*cytb*	cytochrome b
Cytb	Cytochrome b
eCAI	expected CAI
eRCDI	expected RCDI
nAltORF	nested alternative open reading frame
*n**cytb*	nested ORF of *cytb*
Ncytb	Protein of *ncytb* gene
pI	isoelectric point
PTC	premature termination codons
RCDI	Relative Codon Deoptimization Index
SMS	Sequence Manipulation Suite
