# Supplementary material for: Patchy Phylogenetic Distribution and Poor Translational Adaptation of a Nested ORF in the Mammalian Mitochondrial cytb Gene
Source: Genes (Basel). 2025 Jul 17;16(7):833. doi: 10.3390/genes16070833 (PMC12294625; doi:10.3390/genes16070833)
Supplement: Supplementary file 1 [file genes-16-00833-s001.zip › genes-3747614-supplementary/Figure S3 Multiple sequence alignment of cytochrome b nested gene-encoded proteins in Primates.pdf]

```
                      *        20         *
NC_018096. : -MELRLPLRSLPNYSNHHRPILSHTLHPRHSDSLLLSS :  37
NC_019800. : -MKFWLTSWHLLNYSNHHRPIPSHTLHTRYFNRFFLSC :  37
NC_021943. : -MKLWFTSRNLSNPTNYYRPILSNTLLTRHLLCILLNR :  37
NC_021951. : -MKFWFTLRHLFNYSNRHRPIPSYTLYTRHFNRLLFSC :  37
NC_023211. : -MELRLPPRSLPNYPNHHRPILSHTLHPRHPDSLLLSS :  37
NC_026095. : MMKLRLPPRSLSNSSNHYRLISSYTLHSRHHNRILIRS :  38
NC_027449. : -MKLWLTSCSLPNFTNHHRPTPSNTLLTRHLLRLLLNC :  37
NC_027604. : -MKLRLTPYSLPNPTNHHRPILSNTLLTRYLLCLLLNC :  37
NC_034738. : -MKLWFPTRSLSSHPDYHRSIPGHALYSRYNNRVLLRN :  37
NC_037853. : -------------------------------------- :   -
NC_010299. : MMKLWFSPRHLPNPTNLNWVIPRYALYIRYNIRILLHL :  38
NC_012764. : MMKLRLTIRTMPRHPNYHRTILSHTLHTRHNHRILLRH :  38
NC_002811. : MMKLWLSLRGLLRTSNRHRPISSHTLYIRYNNRILLCN :  38
NC_013993. : MMELRLTPWRLPNPPNHHRTIPSHALLTRRLNRLFINR :  38
NC_014047. : MMKLWLTPRRLPSPPNHYRTISSHTLHTRCLRSLLISS :  38
NC_014051. : MMKPWLTPRHLPSPPNRHRVIPGHALYTRRLHSLLISS :  38
NC_008066. : MMKLRLTPRILPNSTDHHRPILSNTLLTRHLFCLLFNR :  38
NC_021957. : MMKPWLTPRHLPNPPNHHRVIPGHALYTRRLHSLLISS :  38
NC_028592. : MMKLWLTSRNLPNPTNYYRFILSNTLLTRHLLCILLNC :  38
NC_011137. : MMKLRLTPWRLPDPPNHHRTIPSHALLTRRLDRLFINR :  38
NC_012920. : MMKLRLTPWRLPDPPNHHRTIPSHALLTRRLNRLFINR :  38
NC_064168. : -MELRLPLRSLPNYSNHHRPILSYTLYLRHSNSLLLSS :  37
NC_064173. : -MELRLPPRSLPNYPNHHRPILSHTLHPRHPDSLLLSS :  37
NC_064182. : -MELRLPLRSLPNYSNHHRPILSYTLHPRHPDSLLLSS :  37
NC_064183. : -MELRLPLRSLPNYSNHHRPILSYTLHPRHPDSLLLSS :  37
NC_064190. : -MKFWLTPWHLLNYSNHHRPIPSHTLHTRYFNRFLLSR :  37
NC_065434. : -MELRFSPRSLPSNPNHYRSISSNTLYSRYNNRVLLRY :  37
NC_084368. : -MKFWFTLRHLFNYSNRHRPIPSHTLYTRHFNRLLFSC :  37
              m                r i s  l  r     l
```

```
             40         *        60         *
NC_018096. : PHHPRRKPRMNNPLHACQRCLHILHMPLPPHWTRPLLR :  75
NC_019800. : PHYPRRQLRLNSPLPTRKWCLHILYLPLSAYRPRLILR :  75
NC_021943. : TYYSRRKLWLDHPLPPRQRRLHTLYLSFPTRRPRPLLW :  75
NC_021951. : PYCPRRKLRMNNPLPTRQRCFHILYLPLPTRRPRLILW :  75
NC_023211. : PHRPRRKPRMNNPLHAYQRCLHILHMPLPPHWTRPLLR :  75
NC_026095. : PHLPRCELRMGNPIPPCQRSIHIFPMSIHPCRPRPLLW :  76
NC_027449. : TYHPRCEIRLNHSLPPRQWCLHTLYLPFPTYRSRPLLW :  75
NC_027604. : TYYPRCELRLNHSLSPRQWCLCTLYLPFPTHRPRPLLR :  75
NC_034738. : PHLPRRKLRLTYPLPPRQRSIHILPMPVYSCRPRSILR :  75
NC_037853. : -------------------------------------- :   -
NC_010299. : PHLPRRKLWLNHSISSCQRSINIFSLPIHPHRTWPVLR :  76
NC_012764. : PRMPRRQLWMTYPLYTCQRSINILHLPIHSHRTRTLLR :  76
NC_002811. : PYLPRRKLWMTYPISTRKRSFYILYMLIHACRPRPLLW :  76
NC_013993. : PYRPRRKLRLNHPLPSRQWRLNILYLPLPTHRTRPILR :  76
NC_014047. : SHYPRRKLWLNHPLSSRQRRLNILHLPIPTYWPRPILR :  76
NC_014051. : PHYPRRKLRLGYPLPSCQRCLNIFHLPIPTHRPGPILR :  76
NC_008066. : THHPRRKPRLNHSLPPCQRRLHIFHLPLSTRRPRSLLW :  76
NC_021957. : PHYPRRKLRLGYPLPSCQRCLNIFHLPIPTHRPGHILR :  76
NC_028592. : THHSRRKLWLDHPLPPRQWRLHTFYLSFSTRRPRPLLW :  76
NC_011137. : PHHSRRKLWLNHPLPSRQWRLNILYLPLPTHRTRPILR :  76
NC_012920. : PHHSRRKLWLNHPLPSRQWRLNILYLPLPTHRARPILR :  76
NC_064168. : PHHPRRKPRMNNPLHACQRCLHILHMPLPSHWTRPLLR :  75
NC_064173. : PHRPRRKPRMNNPLHACQRCLHILHMPLPPHWTRPLLR :  75
NC_064182. : PHHPRRKSRMNNPLHACQRCLHILHMPLPSHWTRPLLR :  75
NC_064183. : PHHPRRKPRMNNPLHACQRCLHILHMPLPSHWTRPLLR :  75
NC_064190. : PYYPRRQLRLNSPLPTRKWCLHILYLPLSTHRPRLVLR :  75
NC_065434. : PHLPRRKLRLNYSISPCQRSIHILFMPIHSCRPRHILW :  75
NC_084368. : PYCPRRKLRMNNPLPTRQRCFHILYLPFPTRRPRLILW :  75
                prr      p    q    i   p      r  l
```

```
                   80         *        100         *
NC_018096. : ILSFSRNLKHRYNSTPHNNSHSIHRLRSPMRPNIIMRS : 113
NC_019800. : IFPLSKDLEYRCYPTTYNHSHSIHRLCPPMRPNIILRG : 113
NC_021943. : LIPPPKNLKYWHHTSSYNYSNSLYGLRTPMRPNIILRG : 113
NC_021951. : ILPFSGDLKRRYYPTTYNYSHSIHRLRPPMGPNIILRG : 113
NC_023211. : ILSFSRNLKHRYNSTSHNNSHGIHGLRSPMRPNIIMRG : 113
NC_026095. : LFYIIRNLKHRYYPVIHSNSYSLHRICPPMRADIILRC : 114
NC_027449. : LIPPPRNLKHWHYTPSYNHNNSFHRLCSPMRPNVILGS : 113
NC_027604. : FIPSSKNLKYRDYTPSYNNDNSFHGLCSSVRPNIILRG : 113
NC_034738. : LLHPARNLKPRHHPTILDNSHSLHRLCPPMRTNIILGR : 113
NC_037853. : -----------------------------MRPNILLRG :   9
NC_010299. : IIHLLRNLKHRNHSPTYSDSNSIYRLCPPMRTNILLRG : 114
NC_012764. : IIQLSRNLKHWNRPIICSNSYRFHGLRPPMRTNIILRR : 114
NC_002811. : LLHLPRNMKHWNPIIIYCNSNRIHRLRTPMRTNIILRR : 114
NC_013993. : IISLLRNLKHRHYSPAYNYSNSLHRLCPPMRPNIILRR : 114
NC_014047. : LIPLPRNLKHWHHPPTHNYSNSLHGLCPPMRTNIFLRS : 114
NC_014051. : LIPLLRNLKHRHYPLISNHSNSLHRLRPPMRPNILLRC : 114
NC_008066. : LIPSPKNLEHWHYTSILDYSNSLYRLRTSMRPNIILRG : 114
NC_021957. : LIPLLRNLKHRHHPLISDHSNSLHRLRPPMRPNILLRC : 114
NC_028592. : LIPPLKNLKYWRHASPHNYSNSLHGLRTPMRPDIILRG : 114
NC_011137. : IISLLKNLKHRHYPPACNYSNSLHRLCPPMRPNIILRG : 114
NC_012920. : IISLLRNLKHRHYPPACNYSNSLHRLCPPVRPNIILRG : 114
NC_064168. : ILSFPRNLKHRYNSTPHNNSHSIHRLRSPMRPNIIMRG : 113
NC_064173. : ILSFSRNLKHRYNSTSHNNSHGIHGLRSPMRPNIIMRG : 113
NC_064182. : ILSFSRNLKHRHNSTPHNNNHSIHRLRSPMRPNIIMRG : 113
NC_064183. : ILSFSRNLKHRYNSTPHNNSHSIHRLRSPMGPNIIMRG : 113
NC_064190. : IFPLSKDLKCRCYPTTYNYSHSIHRLCPPMRPNVILRG : 113
NC_065434. : VLHYTRNLKHRYYPIIYSYSNRFHRICPPMRTNIILRS : 113
NC_084368. : ILPFSGDLKRRYYSTTYNHSHSIHRLRPPMGPNIILRG : 113
                       k         s s h   p6r 16 6r
```

```
                120         *       140         *
NC_018096. : YGDYKPPISHSIYRIQPRRVSLRWLLSRQSHPHTIFYL : 151
NC_019800. : HSNYKPPISHPLHWVQYRTMSLRWLFSRQSYPYTILYL : 151
NC_021943. : NSNHKSTISSPVHRSRYRPMNLRRTRHWQPHPHTILHP : 151
NC_021951. : YSHHKPSISHPLYRVQSCRVNLRWFLSRQSHPYTILYF : 151
NC_023211. : YSDHKPPISHSICRIQPRRMSLRRLLSRQSHPHTIFYL : 151
NC_026095. : HSNHQSTISNPIRWHRSGRMNLRRLLCRQSYTHPILRI : 152
NC_027449. : NSNHKPTIGNPVYRNQSSPMNLRRICHRQPHPHTILHL : 151
NC_027604. : NRNHKLTISNSVYRNQHRPMNLRWVRHWQPYSHTILHP : 151
NC_034738. : YSNYKLTLGNSLHRYRPSRVNLRRLLCRQSYSNPILRT : 151
NC_037853. : HSNHKLAIRHPIHRNKPSPMGLRWLLSRQPHPYTILYL :  47
NC_010299. : HRNHKLTFSNPLYRNQFSRMNLRRILRRQSYPNTIFRL : 152
NC_012764. : HCDYKPPISNPICRHNSSRMNLRRILSRQSHPNTILRL : 152
NC_002811. : YRHHQPSISYPLYWHQSSRMNLRGLLSRQSHPHTIFRL : 152
NC_013993. : HSNYKPTIRHPIHWDRPSSMNLRGLFSRQSHPHTILYL : 152
NC_014047. : HSNHKLIVCHSIHRNKPSPMSLRWLFSRQRHTHTLLHL : 152
NC_014051. : HSNHKPTICRPIHRNRPSPVSLRRLFSGQRYTHTLLYL : 152
NC_008066. : NSDHKPIISNPIHRDRPRPMNLRWVLHWQPHPFTILHP : 152
NC_021957. : HSNHKPTICRPIHRNRPSPMSLRRLFSGQRYTHTLLYL : 152
NC_028592. : NSNHKPTISSTIHRNQSCPMNLRRVRYWQPYPHTILYP : 152
NC_011137. : HSNYKPTIRHPIHWDRPGSVNLRRLLSRQSHPHTILYL : 152
NC_012920. : HSNYKLTIRHPIHWDRPSSMNLRRLLSRQSHPHTILYL : 152
NC_064168. : YSNYKPPISHSICRIQPRRMSLRRLLSRQSHPHTIFYL : 151
NC_064173. : YSDHKPPISHSICRIQPRRMSLRRLLSRQSHPHTIFYL : 151
NC_064182. : YGNYKPPISHSICRIQPRRVSLRRLLSRQSHPHTILYL : 151
NC_064183. : YGDYKPPISHSICRIQPRRVSLRRLLSRQSHPHTILYL : 151
NC_064190. : HSNYKPPISHPLHWVQYRTMSLRWLFSRQSYPYTILYL : 151
NC_065434. : HSHHKLTFSNPIYRYCPSRMNLRGIFSRQSYTNSILCI : 151
NC_084368. : YSNHKSSISHPLYWVQSCRVNLRWFLSRQSHPYTILYF : 151
                 k      6       6 LR    rQ     6l
```

```
                  160         *       180         *
NC_018096. : PLCLTLYHRSTSDYSSIVLT------------------ : 171
NC_019800. : SLYPTLYYCSPSNYPPLVSA------------------ : 171
NC_021943. : ALHPTFHHSRPYSRTLTIST------------------ : 171
NC_021951. : PLYLTLYYCSPSNYSPIISA------------------ : 171
NC_023211. : PLCLTIYHRSTSDYSSIVLT------------------ : 171
NC_026095. : PLYFTLYYYSICHNPPSFST------------------ : 172
NC_027449. : TLYSTLHHHRPYNRTPTIPTRNRIKQPLRNLL------ : 183
NC_027604. : TLHPTLHHRRPHNRTPTLPTRNRIKQPLRNFLQLGYNH : 189
NC_034738. : PFYFTLHYLNLSYNPPSLPSRNGIHQSTRHILKP---- : 185
NC_037853. : PLYFTLHYHSPNNPPSPISTRNRIKQPSRHPLPLRQNY :  85
NC_010299. : PLYPTLHHFNPRNNPSSLSPRIRIQQPIRNFLKLRQNP : 190
NC_012764. : SLHPSLYHYSTSYNSPPISPRNRIKQPIRNSIRIGQNS : 190
NC_002811. : PLYPTFHSNSLSNSPPPIPPRNWIQ------------- : 177
NC_013993. : SLHLALHYCSPSSTPPPILARNRVKQPPRNHLPFR--- : 187
NC_014047. : SLHPTLHHHSPSNPTPTILTRNRIKQPPRHPLPTGQSH : 190
NC_014051. : PLYPALYHHSPSSPAPTILTRNRIKQPPGYLLPTRQNH : 190
NC_008066. : TLYSTFHYHRSYNSPSAIPTRNRIKQPLRNLIRLRQNP : 190
NC_021957. : PLYPALYHHSPSNPAPTILTRNRIKQPPGYLLPTRQNH : 190
NC_028592. : TLYPTFHYSRPYSRTLTISTRNRIKQPLWNPF------ : 184
NC_011137. : SLYLTLHYCSPSSTPPPILARNRIKQPPRNHLPFR--- : 187
NC_012920. : SLHLALHYCSPSNTPPPILARNGIKQPPRNHLPFR--- : 187
NC_064168. : SLCLTLYHRSTSDYSSIILT------------------ : 171
NC_064173. : PLCLTIYHRSTSDYSSIVLT------------------ : 171
NC_064182. : PLCLTLYHRSTSNYSSIILT------------------ : 171
NC_064183. : PLYLTLYHRSTSDYSSIILT------------------ : 171
NC_064190. : SLYSTLYYCSPSNYPSLVSA------------------ : 171
NC_065434. : PLHSTLYYCSPRYSASPISTRNRF-------------- : 175
NC_084368. : PLYLTLYYCSPSNYSPIISA------------------ : 171
              l
```

```
                    200         *       220
NC_018096. : -------------------------------------- :   -
NC_019800. : -------------------------------------- :   -
NC_021943. : -------------------------------------- :   -
NC_021951. : -------------------------------------- :   -
NC_023211. : -------------------------------------- :   -
NC_026095. : -------------------------------------- :   -
NC_027449. : -------------------------------------- :   -
NC_027604. : LSPLLYNQRHPGPNPPSLYPSNASTILTQPPKRPRQLH : 227
NC_034738. : -------------------------------------- :   -
NC_037853. : FPPLLHNQRHPRPIPLSPDPNNTNTILTRPPRRPRQLR : 123
NC_010299. : IPPLLHNKRHLRLDPSLTFPHNPNPILPRPLRRPRQLH : 228
NC_012764. : LPPLLHHQRLTRSCPSPTIPIHPSPFLP---------- : 218
NC_002811. : -------------------------------------- :   -
NC_013993. : -------------------------------------- :   -
NC_014047. : LSPLLYNQRHPRNTPPPPHPNKPSTILTRPPGRPRQLH : 228
NC_014051. : LSPLLHNQRYSRTIPPPSHPNEPSTILARPPR------ : 222
NC_008066. : LPPLLHN------------------------------- : 197
NC_021957. : LSPLLHNQRYSRTIPPPSHPNEPSTILARPPR------ : 222
NC_028592. : -------------------------------------- :   -
NC_011137. : -------------------------------------- :   -
NC_012920. : -------------------------------------- :   -
NC_064168. : -------------------------------------- :   -
NC_064173. : -------------------------------------- :   -
NC_064182. : -------------------------------------- :   -
NC_064183. : -------------------------------------- :   -
NC_064190. : -------------------------------------- :   -
NC_065434. : -------------------------------------- :   -
NC_084368. : -------------------------------------- :   -
```

```
              *       240         *
NC_018096. : ----------------------------- :   -
NC_019800. : ----------------------------- :   -
NC_021943. : ----------------------------- :   -
NC_021951. : ----------------------------- :   -
NC_023211. : ----------------------------- :   -
NC_026095. : ----------------------------- :   -
NC_027449. : ----------------------------- :   -
NC_027604. : SS--------------------------- : 229
NC_034738. : ----------------------------- :   -
NC_037853. : PSQPPKHPTPHQTRMILPICLRNPPICPQ : 152
NC_010299. : PR--------------------------- : 230
NC_012764. : ----------------------------- :   -
NC_002811. : ----------------------------- :   -
NC_013993. : ----------------------------- :   -
NC_014047. : PS--------------------------- : 230
NC_014051. : ----------------------------- :   -
NC_008066. : ----------------------------- :   -
NC_021957. : ----------------------------- :   -
NC_028592. : ----------------------------- :   -
NC_011137. : ----------------------------- :   -
NC_012920. : ----------------------------- :   -
NC_064168. : ----------------------------- :   -
NC_064173. : ----------------------------- :   -
NC_064182. : ----------------------------- :   -
NC_064183. : ----------------------------- :   -
NC_064190. : ----------------------------- :   -
NC_065434. : ----------------------------- :   -
NC_084368. : ----------------------------- :   -
```
