# Supplementary material for: Patchy Phylogenetic Distribution and Poor Translational Adaptation of a Nested ORF in the Mammalian Mitochondrial cytb Gene
Source: Genes (Basel). 2025 Jul 17;16(7):833. doi: 10.3390/genes16070833 (PMC12294625; doi:10.3390/genes16070833)
Supplement: Supplementary file 1 [file genes-16-00833-s001.zip › genes-3747614-supplementary/Figure S4 Multiple sequence alignment of cytochrome b nested gene-encoded proteins in Rodentia.pdf]

```
                          *         20         *
NC_014861. : MMKLRFSPRGMPHNSNHHRPIPSNTLHIRHYNSIFISH :  38
NC_014864. : MMKLRFPPRSMPHNSNHHRPIPSNTLHIRHHNSILISH :  38
NC_014867. : MMELWLSLRDVPHSSNHHRPIPSNTLHIRHHNSILIRH :  38
NC_014871. : MMKLRFSTRSMPHNSNHYRPIPSNTLHIRHHNSILISH :  38
NC_014855. : MMKLRLSPRSMSHNSNHHRTIPSNTLHIRHYNSIFISH :  38
NC_019584. : -MKFWLSTRYLFNCTNYYRSISSYTLYIRYNNSILISH :  37
NC_019585. : -MKFWLSIRYLSNRTNYYRPIPSYTLYIRYNNSILISN :  37
NC_020756. : MMKLRIPTRSLSRIANHYRIIPSNTLHIRHPYSLFISN :  38
NC_023960. : MMKLWLSPRTMPYSTNHYRPFSSNTLHIRHNNGILISH :  38
NC_024592. : MMKLRLPNRTLFNYTNPNRPISSHTLYLRYFNSILISN :  38
NC_025270. : MMKLRLPFRNLPNSPNYYRTIPSYTLYIRYNNSLLISN :  38
NC_025952. : MMKLRISPRNLSNSPNHYRPILSYTLYIRHNDSLLISN :  38
NC_027932. : MMKLRLSPRSVPHRTNYYRIVSSYTLHIRHYNSILISH :  38
NC_028335. : MMEFWLSPRSMPYNSNHYRPVLSNTLYIRHRNSILISY :  38
NC_028625. : -MKLRIPTRNLLNPTNCNRTIPSNTLHIRHHNSIFFSR :  37
NC_029760. : -MKFRIITRNLPSDSNYNRTIPSHTLHIRHINSIFISH :  37
NC_029888. : MMKFWLSTRSMPHNSNYHRPIPSNTLHIRHFNSILISY :  38
NC_030342. : MMKFWLPSRNLPNSPNYYRTIPSYTLYIRYNNSLLISN :  38
NC_033382. : -MKLRLNLRSMLNHPNPYRPISGHTLYLRHYHSIFIRH :  37
NC_033912. : MMKLWIPARNLPNPTNCNRAIPSNTLHIRHRDSILFSR :  38
NC_006914. : MMKLWVPSRSLPNSPNHYRSFLSHTLHIRYNNSLFISN :  38
NC_035593. : -MKLRILTRHLPNNPNPNRTISSNTLHIRYNYSILISN :  37
NC_035598. : MMKLWILTRHLPNNPNSNRIIPSNTLHIRYNHSILISN :  38
NC_035594. : MMKLRITSWSMPSNPNPYRPIPSHTLHIRHFYSILICN :  38
NC_035614. : MMKLRILTRHLPNNSNPNRIIPSHTLHIRYNYSILISN :  38
NC_035615. : -MKLRILTWHLPNYPNPNRTVSSYTLHIRHNHSILISN :  37
NC_035621. : MMKFWLSTRSMPHNSNYHRPIPSNTLHIRHFNSILISY :  38
NC_035822. : MMKLRLPTRTMPYRTNHHRTIPSNTLHIRYNNSILISH :  38
NC_037778. : MMKFWLLTRCMSHITNHYRIIPRHTLYCRYYYSFLISH :  38
NC_039420. : MMKLWLFIRCVFNFTNYYRIISSHTLHRRHNYSLLICC :  38
NC_039547. : MMKLRLFIGCMSNAPDHYRTIPSHALYCRHSHSLLICY :  38
NC_039551. : MMKLRLFIGCMFNSPNHYWTILSHTLYRRHNHSLLICY :  38
NC_040138. : -------------------------------------- :   -
NC_047188. : MMKLRIPTRHLPNNPNPNRTIPSNTLHIRHNYSILISN :  38
NC_048987. : MMKLWIFTRCLLRSPNYYRLIPSHTLYLRYLDSILISN :  38
NC_049040. : MMKFWLSTRSMPHHSNHHRPIPSNTLHIRHFNSILISY :  38
NC_049042. : MMKLRLSTWGMSYNSNHHRPIPSNTLHIRHSNSILISY :  38
NC_049119. : MMKLWFPPRSMPNYPNCNRTISSNTLHIRHHNSIFISN :  38
NC_049121. : MMKLRLPPRNLFNSTNYYRPISSYTLYIRHNNSILISH :  38
NC_049122. : MMKLRLIARNLPDNPNPHRLISSNTLHIRHNNSIFFSD :  38
NC_051518. : -MKFRFTPRNLPNNPNYHRLISSNTLHIRYPNSILISN :  37
NC_051519. : -MKFRFTPRNLPNNPNCHRLIFSNTLHIRYLNSILISN :  37
NC_010650. : -MKLWLPTRNLPNSPNHYRPILSHTLYIRHNNSFLISN :  37
NC_053796. : MMKFRLPTRNLLNSTNHYRPIFSHTLFIRHNNSIFISN :  38
NC_053803. : -MKFWLLTRDLSHTTNCYRTIPSHTLHIRYNNSILISY :  37
NC_053806. : MMKLWLFTRNLLNSTNHYRPIPSHTLYIRYNNSIFISN :  38
NC_053809. : -MKLRIITWNLPNNPNHHRLIPSNTLYIRHNNSIFISH :  37
NC_053813. : -MKLRLPTRNLPDSTNHYRLIPSHTLHIRHNNSILISS :  37
NC_056988. : MMKLWLPFRSMPNHPNHHRTISSNTLHIRHHNSILISH :  38
NC_057104. : MMKFWLSPRSMPYDPNYYRAILSNTLYIRHRNSILISH :  38
NC_011638. : MMKFWLSSRSMPYNSNYHRPIPSNTLHIRHFNSILISY :  38
```

```
NC_012387. : MMKLWIPSRNLPNSSNHHRPFLSHTLYIRYNNSLFISN :  38
NC_012461. : MMKLRFSPRSMPYNSNHHRPIPSNTLYIRHYNSILISH :  38
NC_003041. : -------------------------------------- :   -
NC_036728. : MMKLWLSIRSMPYSPNYDRPISSNTLYIRHHNSIFISR :  38
NC_012374. : MMKFWLSSRSMPYSSNYHRIISSNTLHIRHFNSILISY :  38
NC_035571. : MMKLRILTWSMPNNSNSNRLISSHTLHIRHNYSILISY :  38
NC_002658. : MMKLWIPPRRLLSFTNPYRPISSNTLHSRHNNSIFIRY :  38
NC_006915. : MMKLWVPSRNLPNSPNHHRPFLSHTLYIRYNNSLFISN :  38
NC_012389. : MMKLWLSIRSMPNNSNHYRPVPSNTLHIRHSNSIFISY :  38
NC_027742. : -MELRITPRSLPNPTNYHRSIPIHALHCRHSHRILISS :  37
NC_015112. : -MELRITPRSLPNPTNYHRSIPIHTLHCRHSHRILISS :  37
NC_039921. : MMKLRIPTWSMPNNSNPNRLISSHTLHIRHNYSILISN :  38
NC_023263. : MMKLRISSRYLLNYPNCHRTILSYTLHCRHNNSILISI :  38
NC_025268. : MMKLWLPAGNLLNSPNHYRPIFSYALHIRHNNSFLISN :  38
NC_010339. : MMKLWVPSRNLPNSPNHHRPFLSHTLYIRYNNSLFISN :  38
NC_065079. : MMKLRLTIRNLPNYPNHNRPILSYTLHIRHYNSILISN :  38
NC_065082. : MMKLRLTIRNLPNNPNYHRPLPSNTLHIRHNNSILISN :  38
NC_065337. : MMKFWLSPRIMPHNSDHHRTILSHTLYIRYNNSILISY :  38
NC_065750. : MMKLRLPPRPMPSNPNSYRTIPSHTLHIRHINSILISG :  38
NC_068809. : MMKLRIPTWPMFSNPNPHRLIPGHTLHIRHLHSIFICN :  38
NC_081578. : MMKFWLPTWSLLNYSNPHRIISSYTLHIRYINSILISS :  38
NC_081581. : MMKLRLPPWPLPNHSNPHRTISSHTLHIRHSNSILISG :  38
NC_081583. : MMKFWLTTWPLPDCPNPYRIIPSHTLHIRHINSILISS :  38
NC_081585. : MMKLRLSARTLPNYPNPHRIISSHTLHIRHINSILISS :  38
NC_084043. : MMKFRLLTRHLPSNSNPNRIISSHTLHIRHHHSILISN :  38
NC_085425. : MMKLWVPSWNLPNSPNHHRSILSHTLYIRYNNSLLISY :  38
              mk               r   s tl  r   s  is

             40         *         60         *
NC_014861. : PYLPRCQLRLTDPIFTCQRSLPILHLPIPPRRTRNILR :  76
NC_014864. : PYLPRCKLRLTNSIFTCQWSLYILHLPIPPRRTRYVLR :  76
NC_014867. : PYLPRCKLWLTNPIPTCQRSLHILHLPIPPRRTRNVLR :  76
NC_014871. : PYLPRRKLRLTNPILTCQRSLYILHLPIPPRRTRHVLR :  76
NC_014855. : PYLPRCKLRLTNSILTCQRSLHILYLPVPPCRTRNVLR :  76
NC_019584. : TYLPRRKLRMVNPIYTRKRSLNILYLSISSCRTRSILW :  75
NC_019585. : TYLPRRKLRMINPIYTRKRSLNILHLLIPSRWTRNILW :  75
NC_020756. : SHLPRRKLRLNYPIFTRQRCIHILYLPIPTRRTRYLLW :  76
NC_023960. : PYLPRRKLRLINPLPSRQRSLHILHLLIPPRRTRNILR :  76
NC_024592. : SHLPRCKLWLTYPIPTRQRGFNILYLLIPSRRPRNLLR :  76
NC_025270. : THLPRRKLRMINPIYTRKWSLYILYLLISTCRTRHVLW :  76
NC_025952. : SYLPRRELRVINPIYTRKWSLNIFHLLIPSCRTRNVLR :  76
NC_027932. : PHLSRCKLRLTNPLYTCKWSINIFHLLIYSRRTRHLLR :  76
NC_028335. : PHLPRCELWLINSIPTCQRSFDIFYLPIPSRRTRNVLR :  76
NC_028625. : THLPRRELRLNHPVPSRQRSIHILHLPVPTRRTRNLLR :  75
NC_029760. : TYLPRRKLRMVNSIHTCKRSIYIFHLPFHARRTRNILR :  75
NC_029888. : PHLPRRKLRLTNPILTCQRSFNILYLLIPPCRPRNILR :  76
NC_030342. : PYLPRRKLRMTNPIYTRKWGFNILYLLISTCRTRNILW :  76
NC_033382. : PYLSRRKLWLTNSIFTCQWRFNIFHLPIPTCRSRNILW :  75
NC_033912. : TYLPRRQLRLNYPISSCQRSLHILYLPIHTRRTRGLLW :  76
NC_006914. : THLSRRKLRVTNPIYTRKRSLNIFYLLIPSCRTRLILW :  76
NC_035593. : AYLPRCKLRLINPIHTCKRSFHILYLPIPSCRARNILR :  75
NC_035598. : TYLPRRKLRLTHPIHTRKRSLHILYLPFPPCRTRNILR :  76
NC_035594. : PHLSRRKLWMTYSIYTRKRSFHIFHLLIPTRRTRSILW :  76
```

```
NC_035614. : TYLPRCKLRLTNPIYTCKRGLHILYLLIPSRRTRYILR :  76
NC_035615. : TYLPRRELRLTNPIYTCKRSLHILYLPIPPCRTRNILW :  75
NC_035621. : PHLPRRKLRLINPILTCQRSFNILYLLIPPCRPRNILR :  76
NC_035822. : SHLSRRKLRLIDPILTCKRSFHIFHLLIPSCRTRNILW :  76
NC_037778. : PHLSRRKLWMINPLYTRQRSIYLLYFPLFPYWTRNLLR :  76
NC_039420. : TYLSRRKLRLTNSICTRQWSINILHFTLLPHWSRNLLR :  76
NC_039547. : TYLSRRELWLINSICTRQRSVNILHFPLLSHRTRDLLR :  76
NC_039551. : THLPRRKLWLINSICTRQRSINILHFPLLPYRTRNLLR :  76
NC_040138. : -------------------------------------- :   -
NC_047188. : TYLPRRKLRLTYSIHTRKRSLHILYLPVPTCRPRNILW :  76
NC_048987. : SHLPRRQLRMNHSISTCQRSIHIFHLPFPSCRPRDILR :  76
NC_049040. : PHLPRRKLRLTNSIPTCQRSLNILHLLIPPCRPRNVLR :  76
NC_049042. : SHLSRRKLRLTNPILTCQRSLNILYLLIPPCRSRDILW :  76
NC_049119. : SYLPRRKLRLTNPLYTCKRRLYILYLLISACRTRNILW :  76
NC_049121. : PYLPRRKLRMINPISPCKRSLNIFYLLVPPRRTRNILW :  76
NC_049122. : PYLSRRKLWLTNSIYTCKRSLNIFYLPISSRRTRNVLR :  76
NC_051518. : TYLPRRKLRLTNPIHTRKRSFNIFHLPFSSRRPRNILW :  75
NC_051519. : TYLPRCKLWLTNSIYTCKRSFNIFHLPFSPRRPRNILW :  75
NC_010650. : PHLPRRKLRMTNPIYTRKWSFNIFHLLIFTRRTRNILR :  75
NC_053796. : SHLPRCKLRLIHPIYTCKWSLNILHLPIPTRRPRYILW :  76
NC_053803. : TYLPRRQLWMINSIYTRKRSLNILYLLVSSRRTRNILW :  75
NC_053806. : SHLSRRKLRMINPIYTRKWGLNIFHLPVPPCRTRDILW :  76
NC_053809. : PHLPRCQLRMTHSLHTRQWGLNIFHLFIPTCRTRDVLR :  75
NC_053813. : PYLPRCKLRLTNPIYTCKRSLHILHLPIPPCRTRYILR :  75
NC_056988. : PHLPRRKLRLTNSILTRKRSFHILHLPIPSRRTRHLLR :  76
NC_057104. : PHLSRCKLWLINSISTCQRSLNIFYLSIPSRRTRNVLR :  76
NC_011638. : PHLPRRKLWLTNPILTCQWSLNILYLLIPPCRPRYILR :  76
NC_012387. : THLPRRKLRVTNPIHTRKRSLNIFYLLIPSCRTRLIIW :  76
NC_012461. : PHLPRCKLRLTNSISTCQRSLYILHLPIPPCRTRNVLR :  76
NC_003041. : -------------------------------------- :   -
NC_036728. : TYLPRCKLRLTNPIFTCKRSFYILHLPISSRRTRNILR :  76
NC_012374. : SHLPRRKLRLTNPILTCQRSLNILYLLIPPCRPRDILR :  76
NC_035571. : THLPRRQLRLTYPIHTRKRSLNILHLLIPSCGAWNILW :  76
NC_002658. : SHLSRRKLRMTHPILTRQRSINILYLLIPARSTRYILR :  76
NC_006915. : THLSRRKLRVTNPIYARKRSLNIFYLLIPSCRTRLILW :  76
NC_012389. : PHLPRRKLRLTNSIFTCQRSFNILYLLIPPRRTRNVLW :  76
NC_027742. : PHLPRCKLRMINPILTRQWCLHIFHLPVSSRRSRNILR :  75
NC_015112. : PHLPRCKLRMINPILTRQWCLHIFHLPVSSRRSRNVLR :  75
NC_039921. : THLPRRQLRLTYPIYTRKRSLNILHLLIPSCRTRNILW :  76
NC_023263. : SYLPRRKLRLTYPLHTCQWGLNILYLPFHSYRTRYLLR :  76
NC_025268. : SHLPRRKLRVTNSIYTCKRSLHIFYLLISPRRTRNLLR :  76
NC_010339. : THLSRRKLRVTNPIYTRKRSLNIFYLLIPSCRTRLILW :  76
NC_065079. : THLSRRKLWMINPIYTCKWSINIFHLPFSSCRPRNILW :  76
NC_065082. : TYLPRRKLRMTNSIYTRKRSINILYLPFSSRRTRDILR :  76
NC_065337. : THLPRRKLRMTNSIYTRKRSINILHLLIPTHRPRHILR :  76
NC_065750. : PHLPRRKLRLTNPLHTRQRGLHILHLLVHTRRARNLLR :  76
NC_068809. : PHLPRCKLRMTYPIHARKRSLYILYLFISTRRTRHILR :  76
NC_081578. : PHLPRRKLRLTYPIYACQRSFHIFHLLIPARGTRHLLR :  76
NC_081581. : PYLSRRKLRLTHPLHTCQWSFHILHLPIPTCRPRSLLW :  76
NC_081583. : PYLPRCKLRLTHPIPSCQRSLHILHLLISARRAGNLLR :  76
NC_081585. : PHLSRCKLRLTYTILTCQWSLHILYLFIPTRRTRRILW :  76
NC_084043. : THLPRRQLRVANPIYTRKRSFHILYLPIPPRRTRDILR :  76
NC_085425. : THLSRRKLRVTNPIYTRKRSLNIFYLLIPSCRTRTILR :  76
```

```
                         l r kl            s     l     r r  l

                         80        *        100        *
NC_014861. : ILHFPRDMKYRSYPTICSYSNRIHRLCAPMRANIFLRG : 114
NC_014864. : ILHLPRNMKHWSYPTICSYSNRIHRLRTSMRTNIFLRC : 114
NC_014867. : ILHLPRNMKHRSHSTIHSHSNGIHRLCTSMRTNVLLRG : 114
NC_014871. : ILHLPRNMEHWSYLTICSYSNRIHRLCTSMRTNIFLRS : 114
NC_014855. : ILHLPRNMKHWYYPTIYSHSNRIYRLRTSMRTDIFLRS : 114
NC_019584. : ILHIYRNMKYWGNLTICCDSHSIHRLRSSMRTNIILRC : 113
NC_019585. : ILYIHRNMKHWSYFICSYSYSIYRICTTMRTNIILRS : 113
NC_020756. : VLPLQRNMKHWYYLIIHSNSNRIYRICPPMRTNIILRS : 114
NC_023960. : ILYFPGNMKHWSTSTVRSHSHSIYRLCPSMRTNIILRR : 114
NC_024592. : LLHYSRDMKHRCSPTTYSNSHSLHRICTSLRSNIILRG : 114
NC_025270. : ILYIYRNMKHWSSTTIRSNSHSIYRLCTPMRTNIILRS : 114
NC_025952. : ILHIHRNMKYWSTSTIRSNSHSIYRLCTPMRTNIILRS : 114
NC_027932. : ILRLPRNMKHRSYLTICSNSNRIHRICTPMRTNILLRC : 114
NC_028335. : ILHLSRNMKYWNHSAIRSYSNRIHRLCASMRTNILLRS : 114
NC_028625. : ILHLYRNMKYWYHLTTYSYSHCFHRICTPMRTNILLRC : 113
NC_029760. : IIHLYRNMKHRNYLVICSYSNSIYGVCTSMRTNIFLGG : 113
NC_029888. : ILHFPRNMKHWSRPTICSHSNCIHRLCTPMRTNIILRG : 114
NC_030342. : ILHIHRNMKHWSPPIIRSNSHSIHRLRTAMRTNIILRS : 114
NC_033382. : LLYLHRNLKCRSPLIICSNSNSIHRLRSSMRTNILLRG : 113
NC_033912. : IIYLHRDMEHWHHSTIHSHSNRLYRIRPPVRANILLRG : 114
NC_006914. : IIYIYRNLKHWSTSTVRSHSHSIYRLRPSMRTNIILRC : 114
NC_035593. : LIYIHRNMKHRNCTSICRNSNSIHRLRTPMRTNILLRS : 113
NC_035598. : IIHIHRNMKHRNRTLIRRNSNSIYRLRTPMRTNILLRG : 114
NC_035594. : LIHIHRNMKHRHHSTICRNSNSIHRIRITMRTNILLRS : 114
NC_035614. : LIHIYRNMKHRNHPIICRNSNSIHRLCSPMRTNIILRG : 114
NC_035615. : LIHIHRNMEHWHCTPVRRNSNSIHRLRSPMRTDIILRS : 113
NC_035621. : ILHFPRNMKHWSRPTICSHSNRIHRLRTSMRTNIILRS : 114
NC_035822. : ILHFSRNMEHRSNSSICSYSNCIHRICPPMRTNIFLRR : 114
NC_037778. : IIYLHRNLKYRSYLIIHSYSNSFHRIRPPLRTNIFLRS : 114
NC_039420. : ILHLYRNLKHRSSPTICSNSYCFHRICSSLRTDILLRS : 114
NC_039547. : ILHLHRNLKHRSNSTIRSNSNCFHRICSSLRTNILLRS : 114
NC_039551. : ILYLLRNLKHRSNPTIHSNSNCFHRIRSSLRTNVLLRS : 114
NC_040138. : -------MKHRNYPTVCCYGNSLHRLCTPMRSNIILRS :  31
NC_047188. : LIHIHRNMKYRNFTLIRRNSNSIHGLRTSMRTNIILRS : 114
NC_048987. : IIQLLRNMKYRRHLIIRCNSNSIHRICSSMRTNIILRS : 114
NC_049040. : VLHFPRNMKHWSCPTICSHSNRIHRLCTPMRTNIILRG : 114
NC_049042. : ILHFPRNMKHWNCPTICSHSNRIHRLCPSMRTNIILRS : 114
NC_049119. : ILHLHGNMKHWSYSTIRSNSNCIHRLRTSMRTNILLRG : 114
NC_049121. : ILYLPRNMEHWSNPPIRSYSYSIHRLCAPMRTNIILRG : 114
NC_049122. : IIYFYRNMKHWCSSSIRSNSHSIHRICSSMRTNILLRS : 114
NC_051518. : ILHLHRNMKYWSNPTIRSNSYRIHRLCTSMRTNIILRG : 113
NC_051519. : ILHLYRNMKYWSNSTIRSNSYCIHRLCAPMRTNIILRS : 113
NC_010650. : ILYIYRNMKHWSPSIICSNSHSIHRLCTTMRTNIILRS : 113
NC_053796. : ILHILRNMKHWSATIIYSHSHCIHRVCAPMRTNIILRS : 114
NC_053803. : VLHIPRNMEYRCATIIHRHSHRIHRLRPSMGTNIILRR : 113
NC_053806. : ILYISRNMKHWNYSTIYSYSHRIYRICTSMRTNILLRG : 114
NC_053809. : ILHIPRNMKYWSNLIICSYSNCIYRLRPTMRTNIILRG : 113
NC_053813. : ILHISRNMKHWSNPIIYSYSHCIHRLCTPMRTNIILRC : 113
NC_056988. : LLRLHRNMKHRSHSIICSYSNRIHRLRTPMRTNILLRC : 114
NC_057104. : ILHLSRNMKYWNHSAIRSYSNRIHRLCTSMRTNILLRS : 114
```

```
NC_011638. : ILHLFRNMKHWSCPIICSHSNRIHRLCTSMRTNIILRG : 114
NC_012387. : IIYIYRNLKHWSTSTVRSHSHSIYRLCPSMRTNIILRR : 114
NC_012461. : ILRLPRNMKHRSYPTIRSYSNRIHRLCTSMRTNILLRR : 114
NC_003041. : -------MKYRDYPTIRRNSDSIHRLCTSMRTNIILRS :  31
NC_036728. : ILHFLRNMKHWSNPTISNHSNRIHRLCTSMRTNILLRG : 114
NC_012374. : ILHLLRNMKHWNYPTICSHSNRIHRLCTPMRTNIILRG : 114
NC_035571. : IIHIHRDMKHRSCTIICCNSNSIHRICTSMRTNILLRS : 114
NC_002658. : IIHISRNLKFRSNSTTFCYSNRFHRIRSSLRTNIFLRC : 114
NC_006915. : IIYIYRNLKHWSTSTVRSHSHSIYRLRPSMRTDIILRR : 114
NC_012389. : ILHLPRNMKHWNRPTICSNSHRIHRLRTSMRTDIILRS : 114
NC_027742. : ILHIHRNLKHRNHSSPISYSNCFHRICATMRTNIILRC : 113
NC_015112. : ILHIHRNLKHRNHSSPISYSNCFHRICTTMRTNIILRC : 113
NC_039921. : VIHIHRNMKHWSCTIICCNSNSIHRICTSMRTNILLRS : 114
NC_023263. : ILYLYRNMKLRSNPFIHCNSYCIYRLRPSMRANILLRS : 114
NC_025268. : ILCIYRNMKYWSPPFIRSNSYSVHRLCSAMRTNIILRC : 114
NC_010339. : IIYIYRNLKHWSTSTVRSHSHSIYRLRPSMRTDIILRR : 114
NC_065079. : IIHIYRNLEHWGNSTFCSNSNCIHRLCTPMRTNIILRG : 114
NC_065082. : ILHLYRNVKHRSSPTFHSHSHCIHRLCTPMRTNIVLRC : 114
NC_065337. : ILYLYRNMKHWNHSTIYRNSNRIHRIRPSMRTNIILRS : 114
NC_065750. : FIQHNRNMKHRHYFTIRCYSNSIHRLRPTMRTNIILRR : 114
NC_068809. : LIHIYRNMKHRYYPIIRRHSNSIYGICFTMRTNILLRS : 114
NC_081578. : LLQHNRNMKHRHHPTIRRHSHSIHRLCTPMRPNIILRR : 114
NC_081581. : LLQYSRNMKYRNYPSIRRNSHSIHRLRTPMGPNIILRG : 114
NC_081583. : FLQYNRNMKHRHYPAIRCHSHSIHRLRSSMRPNIILRS : 114
NC_081585. : ILQHNRNMKHRYCPIIRRHSHSIHRLRTPMRPNIILRC : 114
NC_084043. : LILIHRNMKHRNYSIICRNSNSIYRLRTSMRTNILLRG : 114
NC_085425. : ILYIHRNLKHWSAPTIRSNSHSIHRLCPSMRTNIILRS : 114
                  r 6k          s    r6   6r 16 Lr

                 120       *        140       *
NC_014861. : HSNYKPTISYPLHRNYLSRMNLRRLLSRQSNFNPFFRL : 152
NC_014864. : HSNYKSTISYPLYRNHPSRMNLRRLLSRQSNLNSFFHL : 152
NC_014867. : HGNYKPSISYSLHRNHLGRMNLRGLLSRQSNLNPFLRF : 152
NC_014871. : HSNYKSTISYPLYRNHPSRMNLRRLLSRQSNLNSFFRL : 152
NC_014855. : YSNHKPTISYPLHRNHPSRMNLRGLLSRQSNFNPFLRL : 152
NC_019584. : NRNYKSSISHSIYRNYPSRMNLRGILSRQSYLDTLFRL : 151
NC_019585. : NSYYKPFISYPIHRNNTSRMNLRWILSRQSHFNTFFRI : 151
NC_020756. : YGHYKSIISYPIRRNNPSRMNLRRFLSRQSHTHPIFRL : 152
NC_023960. : HSYYKPPISHPIYWNNPSRMNLGRLLSRQSHLDPILRF : 152
NC_024592. : NCNYQPFISNPIHRNNLSRMNLRGLLSRQSNLNPILRI : 152
NC_025270. : NSNYKPFISYSLHRNNPCRMNLRWILSRQSHFNPILRL : 152
NC_025952. : HSYYKPPISYPIYWNNPSRMDLRGLLSRQSYPNPILRL : 152
NC_027932. : NSYYQPTISSPICRNYPSRMNLRRILCRQSNFNTILCI : 152
NC_028335. : NSNYKPIISYPLHWLYPSRMNLRWFLCRQGNPNTLLRI : 152
NC_028625. : HSNHKPTLSNPIYRIQPSRMNLRRLFRRQSNPNPILRI : 151
NC_029760. : HSNHKPPISYPLHRNNTSRMNLRGVFSRQSNINTILRI : 151
NC_029888. : HSNHKPIISHSLHWYHLSRMNLRRLLSRQSNPNTFFRI : 152
NC_030342. : HSNYKSFISYSLHRNDPSRMNLRWLLSRQSHFNPILRL : 152
NC_033382. : YSHYKPSFRYPLYWHYLSRMNLRRFLRRQSYFNTILCL : 151
NC_033912. : HSNHKSTLSNSIHRPQPSRMNLRRLLCRQSHPNPILCT : 152
NC_006914. : HSYYKPPISHPIYWNNPSRMNLRGLLSRQSHLDPILRF : 152
NC_035593. : HSNYQPPISHPIHRNNLSRMNLRGILSRQSYPYPILRI : 151
NC_035598. : HSNYQPPISHPLHRNNPSRMNLRRILSRQSHPNPILRI : 152
```

```
NC_035594. : HRNYKPPISYPLCRNYPSRVNLRRFLSRQGHSNTLLRL : 152
NC_035614. : YSNYQSSIGYPLCRNNLSRMNLRRIFSRQSNPNTILRI : 152
NC_035615. : HSNYQPPISYSLYRNNTSRVNLRRILSRQSNPNPIFRI : 151
NC_035621. : HSNYKPIISYSLHWYHPSRMNLRRLLSRQSNPNTFFRI : 152
NC_035822. : HSNHKPSISNSLHRNNPSRMNLRRLLSRQSNLNTFFRI : 152
NC_037778. : NSYYKLIISHPLHWPYPGRMNLRWVFSRQSNFNPILCL : 152
NC_039420. : NSHHKSPLSYPLYRSYSRRMNLRGIFSRQSNPYTILRL : 152
NC_039547. : NSHHQSTLSYSLYWSYPRRMNLRRLLSRQSNPYTILRL : 152
NC_039551. : NSHYQPALSHPLHWPRPRRMNLRGLLSRQSNPYTILRL : 152
NC_040138. : HSNYKPPVSYPLYRLNPGRVNLRRLFSRQGHPHTILRL :  69
NC_047188. : HSNYQPPISHPLHRDNLSRMNLRRILSRQSHPNPILRI : 152
NC_048987. : YSHYQSFISYPIRRNLPSRMNLRWIFSRQSYPNTLLRI : 152
NC_049040. : HSNHKPIISHPLYRHHPSRMNLRGLLSRQSNSNTFLRI : 152
NC_049042. : HSYHKPIISYSLHWHNPGRMNLRRLLSRQSNSNTLFRI : 152
NC_049119. : NSNHKPLISYPIYRNYPSRMNLRGFLSRQSYPNTLLCI : 152
NC_049121. : NSNYKFTFRYSIHRHHTSRMNLRGLFSRQSHTNTILRI : 152
NC_049122. : YSNYKSTISNSIHRNYPSRMNLRRILSRQSYIDTFFRL : 152
NC_051518. : NSNYKLTFSNSICWDNLSRMNLRRIFSRQSNTNTFLCI : 151
NC_051519. : NSNYKLTLSNPIYRNNLSRMNLRRVFSRQSNTNTFLRI : 151
NC_010650. : YSNYKPPISNPLHRNNLSRMNLRWLLSRQGYPNPIFCL : 151
NC_053796. : HSNYQPTLSHPIHRNNPSRMNLGWFLSRQSHPHSIFHI : 152
NC_053803. : NSNYESTLSHPIHWHCPSRMNLRRILRRQSYFNPFLRI : 151
NC_053806. : NCNYKSTLSHPLYRIYSSRMNLRRFLSRQSNSNSFFRI : 152
NC_053809. : NRNHKPAFSYPIHRDHFSRMNLRRLLSRQSHFNPIFRI : 151
NC_053813. : YSNYKPAISHPIHRNHPSRMNLRGFLSRQSNPNPFLCI : 151
NC_056988. : NSNYKPSIGYSIHRNHSSRMNLRRLLSRQGYPDTFFRI : 152
NC_057104. : NSNYKSIISYPLYWLYPSRMNLRWFLCRQGNSNTLFRI : 152
NC_011638. : HSNHKPIISYSLYWHHPSRMNLRRLLSRQSNPNTFFCI : 152
NC_012387. : HSYYKPPISHPIYWNNPSRMNLGRLLSRQSHLDPILRF : 152
NC_012461. : HSNYKSSISYPLYRNYPSRMNLRRLLSRQSNPNSLFRL : 152
NC_003041. : HSNHKPTISYPIHRHNPSRMNLRRILSRQSYPHTILRL :  69
NC_036728. : YSNHQPIISYSLYRNYPSRMNLRRLLSRQSNPNTFFRI : 152
NC_012374. : HSNHKPIISHSLHWHHSSRMNLRRLLSRQSNPNTFFRI : 152
NC_035571. : HSNYQPTISYPLYRNNPSRVNLRGILSRQSNPNTILRI : 152
NC_002658. : NCHHKSPLSHPIHRTYPSRMSLGRILCRQSNLNPIFRP : 152
NC_006915. : HSYYKPPISHPIYWNNPSRMNLRGLLSRQSNLDPILRF : 152
NC_012389. : HSNYKSTISYSLYRHYPSRMNLRGILSRQSNPNSLFRI : 152
NC_027742. : DSHHKPILSNPLHRPNTSRMNLRRIRSRQSHSDPILRL : 151
NC_015112. : HSHHKPILSNPLHWPNTSGMNLRRIRSRQSHSDPILRL : 151
NC_039921. : HSNYQPIISYPLYRNNLGRVNLRRILSRQSNPNTILRI : 152
NC_023263. : HSHYQSSLSNSIYRSNNCRVNLRGVFRRQSNPNTILRI : 152
NC_025268. : NSNYKSFISYSIYRYNPSRMDLRWVLSRQSYTNPIFCF : 152
NC_010339. : HSYYKPPISHPIYWNDPSRMNLRGLLSRQSNLDPILRF : 152
NC_065079. : NSHHKPTISNPIHRNYPSRMNLRWILSRQSNTNTFLCI : 152
NC_065082. : NSNYKPIISNPIHRNHPSRMNLRGILSRQSNTNTFLCI : 152
NC_065337. : NRYYQPTISHPIHRNNPSRMNLRRIFSRQSHPNTIFRP : 152
NC_065750. : HSNYKPPISHPLHRHNTSRMDLRRFLSRQSHSYTILCF : 152
NC_068809. : NRNHKPPISYPLHRNHPSRMNLRRVLSRQSYTNTLLRF : 152
NC_081578. : HSNYKSFISHPLHRNNPSRVNLRRLLSRQSHPHTILCF : 152
NC_081581. : YSNHKPLISYPIHRHNPSRMNLRRLFRRQSHPHTILCL : 152
NC_081583. : HSNYKPTISYPLHRHIPSRMNLRRVLSRQSHPHTIFCL : 152
NC_081585. : HSNYKPPISHPIHRYNPSRMNLRGFLSRQSHPNTILRL : 152
NC_084043. : YSNYQPTLSRPLHRNNSSRMNLRWFLSRQSNPNTIFRI : 152
```

```
NC_085425. : YSYYKPPISYSIHWNNPSRMNLRRLLSRQSYPDSILRL : 152
              s      s  6     sr6 Lr  lsRQs

                  160         *       180         *
NC_014861. : PLHSPIYYRRSCNCTSPFSS------------------ : 172
NC_014864. : PLHSPIYHCRPCNSTSPFSSRNRIK------------- : 177
NC_014867. : PLHPPIYHRRPCNRPPPLSTRNRIKQPHRTRLQRRQNS : 190
NC_014871. : PLHSPIYHCRSCNCPSPFSSRNRIKQPHRTKLQLRQNP : 190
NC_014855. : PLHPPVYHRCPCDRASPFSTRNRIK------------- : 177
NC_019584. : PLYPTIYHRSPSNRSLTIPS------------------ : 171
NC_019585. : SFYSPIHHRSPSSRPSTFPTRNWLKQPNRPKLRRG--- : 186
NC_020756. : PLHPTIHHHSPSGSSPFIPS------------------ : 172
NC_023960. : PLHLTIYHRGPSNRSPSFPSRNRIKQPNRIKLRCR--- : 187
NC_024592. : PLYFTIHHHCTSTSSSLIPTRNRLQQPFRPQFRRRQNP : 190
NC_025270. : PLYSTIHYCSHSNCTSSISPRNRIKQPNRLKLRRRQNP : 190
NC_025952. : PLHLAIHHRSPSNRTPPLSP------------------ : 172
NC_027932. : PLYPSLHHRSPSSCPSTFPSRNRF-------------- : 176
NC_028335. : PLHPPIYHRRTCNRTSPLPSRNRIKQPHRARLQCR--- : 187
NC_028625. : PLHLTLHHHSTSNSSPPILTRNRIKQPIRN-------- : 181
NC_029760. : PLHSTIYHYSPSSCTSSIPP------------------ : 171
NC_029888. : PLHPPIHYRRPCNCTSPLPPRNRIKQPHRTKL------ : 184
NC_030342. : PLYSTIHYCSHSNCTSSIPSRNRIKQPYRPKLRRRQNP : 190
NC_033382. : PFHPSFCNRSLSNSTLNLPS------------------ : 171
NC_033912. : PLHLTLYYCSTGNSPPPIPTRNRIQ------------- : 177
NC_006914. : PLHLTIYYRGPSNRSPPLPPRNRIKQPNRIKLRCR--- : 187
NC_035593. : PLHPTIHHYSSCSSPPTIFTRNRIQ------------- : 176
NC_035598. : PLHPTIHHYSSRSSTSTIPT------------------ : 172
NC_035594. : PLHPSIHYHSPRRSTPTISPRNRI-------------- : 176
NC_035614. : PLHPTLHHHSPRSSPSSISTRNRIKQPIRIKL------ : 184
NC_035615. : SLHSTIHYHSPSSSTSTISTRNRI-------------- : 175
NC_035621. : PLHPPIHHRRPCNCTSPLPPRNRIKQPHRTKL------ : 184
NC_035822. : PFHSPIHHCSSSNCPPSIPSRNRIKQPNRTKLRRR--- : 187
NC_037778. : SLRTPIHYYSNSNNSFIISTRNRI-------------- : 176
NC_039420. : PFRITFYHHSNSHNSLVIPP------------------ : 172
NC_039547. : PLRTPLYHHSNSNNSLTIPSRNRIQQSLRTKLKLGQNP : 190
NC_039551. : PLRTSFYYYSNSNNSLTIPSRDRIQQPFRTKLRLR--- : 187
NC_040138. : PLHPPLYYHRPRASPPSIPTRDRIQQPNRIKLRYRQNP : 107
NC_047188. : PFHFTIHHHSPRSSTSAIPT------------------ : 172
NC_048987. : PFYSSIYYHCLSNCSSPIPTRNRIKQPYRIKFRCRQNS : 190
NC_049040. : PFYPPIHYRRPCNRTSPLPPRNRIKQPHRTKLRRRQNS : 190
NC_049042. : PLYPPIYHRRPCNRTSPLPPRNRIKQPHRTKL------ : 184
NC_049119. : PFYSPIHHCSSCNCPLIISSRNRLK------------- : 177
NC_049121. : PLYSSIHHHSPRNCPPTFPPRNRLKQPYRS-------- : 182
NC_049122. : SLYPSIYYCCPSNCSPLVSP------------------ : 172
NC_051518. : SLYPTIHHHGPSYCPPSIPPRNWIQQPYRPKLRLRQNS : 189
NC_051519. : SLYPTIHYRGPSYCPPSIPPRNWIQQPYRPKLRLRQNS : 189
NC_010650. : PLHLTIHHYSYSNCSPPIPTRNRI-------------- : 175
NC_053796. : SLYLALHHRSSCNSPFTIPTRNRL-------------- : 176
NC_053803. : SLHPSIYHRSPRHCPPPIPS------------------ : 171
NC_053806. : PFHSTIHHYSSCNCSSTFPP------------------ : 172
NC_053809. : PLHPAIHHYSPSNCPLTVSPRNRLKQPHRPKLRRR--- : 186
NC_053813. : PLYFTIHHHSSCNRTPTIPTRNRLKQPNRPKLRRRQNS : 189
NC_056988. : PLHPPIYHCSPSNCSSFISPRNRFKQPHRLKFRRRQNP : 190
```

```
NC_057104. : PLHPPIYHRRTCNRTSPLPSRNRIKQPHRVRLQRR--- : 187
NC_011638. : PLHPPIYHRRPCNCTSPLPPRNRIKQPHRTKL------ : 184
NC_012387. : PLHLTIYHRGPSNRSPSFPSRNRIKQPNRIKLRCR--- : 187
NC_012461. : PLHSPIYHRRPCNCTSPFPTRNRIKQPHRTRLQRRQNP : 190
NC_003041. : PLHPTIYYYRPSTSTPIIPPRNRIQQPNRTKLRLRQNS : 107
NC_036728. : PLHSSIYHCCPRNCTSPFPTRNRIKQPHRTKPQRRQNS : 190
NC_012374. : PLHPPIHHRRPCNCTSPLPPRNRIKQPHRTKL------ : 184
NC_035571. : SLHPTIHHHSPSSSSPIIPA------------------ : 172
NC_002658. : PFHPSLHYHRPNNSSPTIPS------------------ : 172
NC_006915. : PLHLTIYHRGPSNRSPSFPPRNRIKQPNRVKLRCR--- : 187
NC_012389. : PLHSPIHHRRSCNRTSPFPPRNRIKQPHRSKF------ : 184
NC_027742. : PLHPTVHHHSTYDSTPAIPTRNWIKQPIRHQLRLGQNP : 189
NC_015112. : PLHPTIHHHSTYNSTPAIPTRNWVKQPIGHQLRLGQNP : 189
NC_039921. : PLHLTFHHHSPSSSPPIIPT------------------ : 172
NC_023263. : SFYPTIYHRSLSFSPPFIFT------------------ : 172
NC_025268. : PLHSTIYHCSPSNCPPSIPTRNRVKQPNRIKLRRRQNP : 190
NC_010339. : PLHLTIYHRGPSNRSPSFPPRNRIKQPNRVKLRCR--- : 187
NC_065079. : PLHPPIYHHSPSHRSSPISSRNRI-------------- : 176
NC_065082. : SLYPSIYYYSSSLRTPIISPRNRF-------------- : 176
NC_065337. : PLYSPIHYHRPSSSPSPIPTRNRLQQPNRI-------- : 182
NC_065750. : PLHFAFHHHRPCISTSTIPTRNRIQQPNRTELRCRQNP : 190
NC_068809. : PLHPPIYYYSHSCSPPAIPSRNRVQQPNRD-------- : 182
NC_081578. : PLHPTLYYHCSSISSPFIPTRNRLQQPNWTRLRRRQNP : 190
NC_081581. : PLHPTFHYRRSSTCPPIIPARNRVQQPNRTELRYRQNP : 190
NC_081583. : PLHLTLYHHRPRASPPTISTRNRIQQSNWTELRRRQDP : 190
NC_081585. : SLHPTLYHYRPRASSPTIPT------------------ : 172
NC_084043. : PLYSPIHHHSTSSSPPFIPTRNRIQQPIRT-------- : 182
NC_085425. : PLHLTIYHRGPSDRSPSFPPRNRIKQPNRIKLRRR--- : 187
             pl

                   200         *       220
NC_014861. : -------------------------------------- :   -
NC_014864. : -------------------------------------- :   -
NC_014867. : IPPLLHN------------------------------- : 197
NC_014871. : IPPILHN------------------------------- : 197
NC_014855. : -------------------------------------- :   -
NC_019584. : -------------------------------------- :   -
NC_019585. : -------------------------------------- :   -
NC_020756. : -------------------------------------- :   -
NC_023960. : -------------------------------------- :   -
NC_024592. : IPSLLHNQRFPRSPPPFNSSRNFSIVFPRCPRRPR--- : 225
NC_025270. : IPPLLHN------------------------------- : 197
NC_025952. : -------------------------------------- :   -
NC_027932. : -------------------------------------- :   -
NC_028335. : -------------------------------------- :   -
NC_028625. : -------------------------------------- :   -
NC_029760. : -------------------------------------- :   -
NC_029888. : -------------------------------------- :   -
NC_030342. : IPPLLHYQRHFRSHHHISISHDFSTILSRHIRRSRQLH : 228
NC_033382. : -------------------------------------- :   -
NC_033912. : -------------------------------------- :   -
NC_006914. : -------------------------------------- :   -
NC_035593. : -------------------------------------- :   -
```

```
NC_035598. : -------------------------------------- :   -
NC_035594. : -------------------------------------- :   -
NC_035614. : -------------------------------------- :   -
NC_035615. : -------------------------------------- :   -
NC_035621. : -------------------------------------- :   -
NC_035822. : -------------------------------------- :   -
NC_037778. : -------------------------------------- :   -
NC_039420. : -------------------------------------- :   -
NC_039547. : ISPLLHN------------------------------- : 197
NC_039551. : -------------------------------------- :   -
NC_040138. : ISPILYNQRPAGGPLLINSSHNFGFIFPRCARRPRQFY : 145
NC_047188. : -------------------------------------- :   -
NC_048987. : ISPLLYY------------------------------- : 197
NC_049040. : ISSILHN------------------------------- : 197
NC_049042. : -------------------------------------- :   -
NC_049119. : -------------------------------------- :   -
NC_049121. : -------------------------------------- :   -
NC_049122. : -------------------------------------- :   -
NC_051518. : IPPLLHNQRHSRSNLNIHVTSNSSSILPRPTRRSR--- : 224
NC_051519. : IPPLLHNQRHPRSNFNIHIPNNPSIILPRPTRRSR--- : 224
NC_010650. : -------------------------------------- :   -
NC_053796. : -------------------------------------- :   -
NC_053803. : -------------------------------------- :   -
NC_053806. : -------------------------------------- :   -
NC_053809. : -------------------------------------- :   -
NC_053813. : ISPILHH------------------------------- : 196
NC_056988. : IPSLLYNQRPPRNFHPPPLPNNLSPILPRPPRRPRQLY : 228
NC_057104. : -------------------------------------- :   -
NC_011638. : -------------------------------------- :   -
NC_012387. : -------------------------------------- :   -
NC_012461. : IPSILHN------------------------------- : 197
NC_003041. : IPPLLHNQRPLRCHHLIDSFHNFGFIFPRYSRRPRQLH : 145
NC_036728. : ISPILHN------------------------------- : 197
NC_012374. : -------------------------------------- :   -
NC_035571. : -------------------------------------- :   -
NC_002658. : -------------------------------------- :   -
NC_006915. : -------------------------------------- :   -
NC_012389. : -------------------------------------- :   -
NC_027742. : IPPLLLI------------------------------- : 196
NC_015112. : IPPLLLI------------------------------- : 196
NC_039921. : -------------------------------------- :   -
NC_023263. : -------------------------------------- :   -
NC_025268. : IPPLLHNQRYLRYPYHIPIPYNFSIIFPRYIRRSR--- : 225
NC_010339. : -------------------------------------- :   -
NC_065079. : -------------------------------------- :   -
NC_065082. : -------------------------------------- :   -
NC_065337. : -------------------------------------- :   -
NC_065750. : IPPVLHN------------------------------- : 197
NC_068809. : -------------------------------------- :   -
NC_081578. : IPPLLHNQRLLRGPRPINSSHNFGFIFPRYSRRPRQLH : 228
NC_081581. : IPPLLHNQRLPRHPYPINSFHNFGFIFPRYSRRPRQLH : 228
NC_081583. : LPPLLYNQRLLRGFITINSSHNFGFIFPRCSRRP---- : 224
NC_081585. : -------------------------------------- :   -
```

```
NC_084043. : -------------------------------------- :    -
NC_085425. : -------------------------------------- :    -


              *       240         *
NC_014861. : ---------------------------- :    -
NC_014864. : ---------------------------- :    -
NC_014867. : ---------------------------- :    -
NC_014871. : ---------------------------- :    -
NC_014855. : ---------------------------- :    -
NC_019584. : ---------------------------- :    -
NC_019585. : ---------------------------- :    -
NC_020756. : ---------------------------- :    -
NC_023960. : ---------------------------- :    -
NC_024592. : ---------------------------- :    -
NC_025270. : ---------------------------- :    -
NC_025952. : ---------------------------- :    -
NC_027932. : ---------------------------- :    -
NC_028335. : ---------------------------- :    -
NC_028625. : ---------------------------- :    -
NC_029760. : ---------------------------- :    -
NC_029888. : ---------------------------- :    -
NC_030342. : TSQSTQHPTPH----------------- :  239
NC_033382. : ---------------------------- :    -
NC_033912. : ---------------------------- :    -
NC_006914. : ---------------------------- :    -
NC_035593. : ---------------------------- :    -
NC_035598. : ---------------------------- :    -
NC_035594. : ---------------------------- :    -
NC_035614. : ---------------------------- :    -
NC_035615. : ---------------------------- :    -
NC_035621. : ---------------------------- :    -
NC_035822. : ---------------------------- :    -
NC_037778. : ---------------------------- :    -
NC_039420. : ---------------------------- :    -
NC_039547. : ---------------------------- :    -
NC_039551. : ---------------------------- :    -
NC_040138. : SCKSTQHSTTH----------------- :  156
NC_047188. : ---------------------------- :    -
NC_048987. : ---------------------------- :    -
NC_049040. : ---------------------------- :    -
NC_049042. : ---------------------------- :    -
NC_049119. : ---------------------------- :    -
NC_049121. : ---------------------------- :    -
NC_049122. : ---------------------------- :    -
NC_051518. : ---------------------------- :    -
NC_051519. : ---------------------------- :    -
NC_010650. : ---------------------------- :    -
NC_053796. : ---------------------------- :    -
NC_053803. : ---------------------------- :    -
NC_053806. : ---------------------------- :    -
NC_053809. : ---------------------------- :    -
NC_053813. : ---------------------------- :    -
```

```
NC_056988. : TSKPPKHTTPY----------------- : 239
NC_057104. : ---------------------------- :   -
NC_011638. : ---------------------------- :   -
NC_012387. : ---------------------------- :   -
NC_012461. : ---------------------------- :   -
NC_003041. : SCKSTQYPTTHQTRMIFSLRLRHLTIHP : 173
NC_036728. : ---------------------------- :   -
NC_012374. : ---------------------------- :   -
NC_035571. : ---------------------------- :   -
NC_002658. : ---------------------------- :   -
NC_006915. : ---------------------------- :   -
NC_012389. : ---------------------------- :   -
NC_027742. : ---------------------------- :   -
NC_015112. : ---------------------------- :   -
NC_039921. : ---------------------------- :   -
NC_023263. : ---------------------------- :   -
NC_025268. : ---------------------------- :   -
NC_010339. : ---------------------------- :   -
NC_065079. : ---------------------------- :   -
NC_065082. : ---------------------------- :   -
NC_065337. : ---------------------------- :   -
NC_065750. : ---------------------------- :   -
NC_068809. : ---------------------------- :   -
NC_081578. : TCKSTQYSTTH----------------- : 239
NC_081581. : SCKSTQHSTPH----------------- : 239
NC_081583. : ---------------------------- :   -
NC_081585. : ---------------------------- :   -
NC_084043. : ---------------------------- :   -
NC_085425. : ---------------------------- :   -
```
