# Supplementary figures and images for: Patchy Phylogenetic Distribution and Poor Translational Adaptation of a Nested ORF in the Mammalian Mitochondrial cytb Gene
Source: Genes (Basel). 2025 Jul 17;16(7):833. doi: 10.3390/genes16070833 (PMC12294625; doi:10.3390/genes16070833)

Tree scale: 0.1

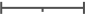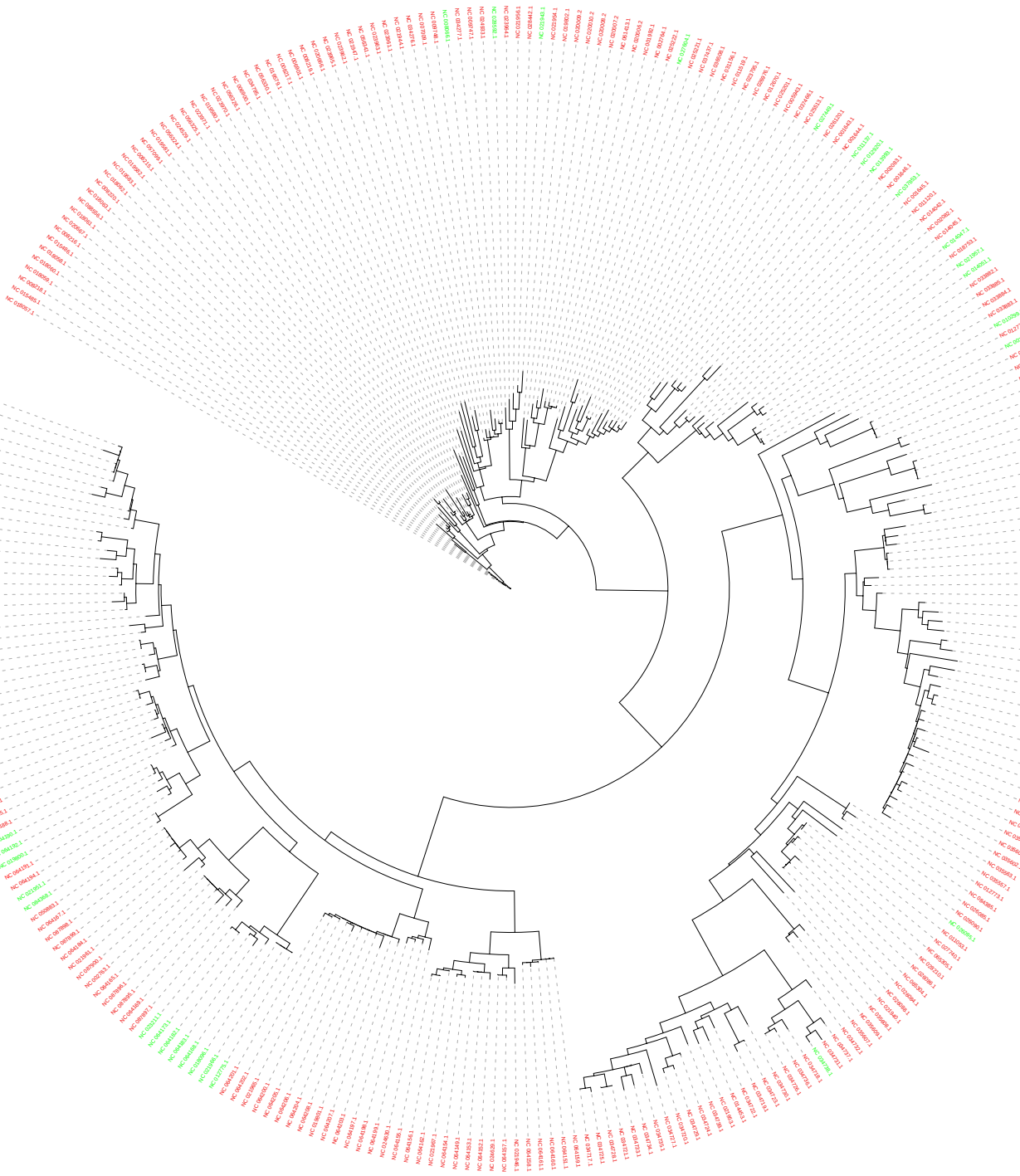

Supplement: Supplementary file 1 [file genes-16-00833-s001.zip › genes-3747614-supplementary/Figure S1 Phylogenetic tree of cytochrome b encoded proteins in Primates.pdf]

Tree scale: 0.1

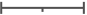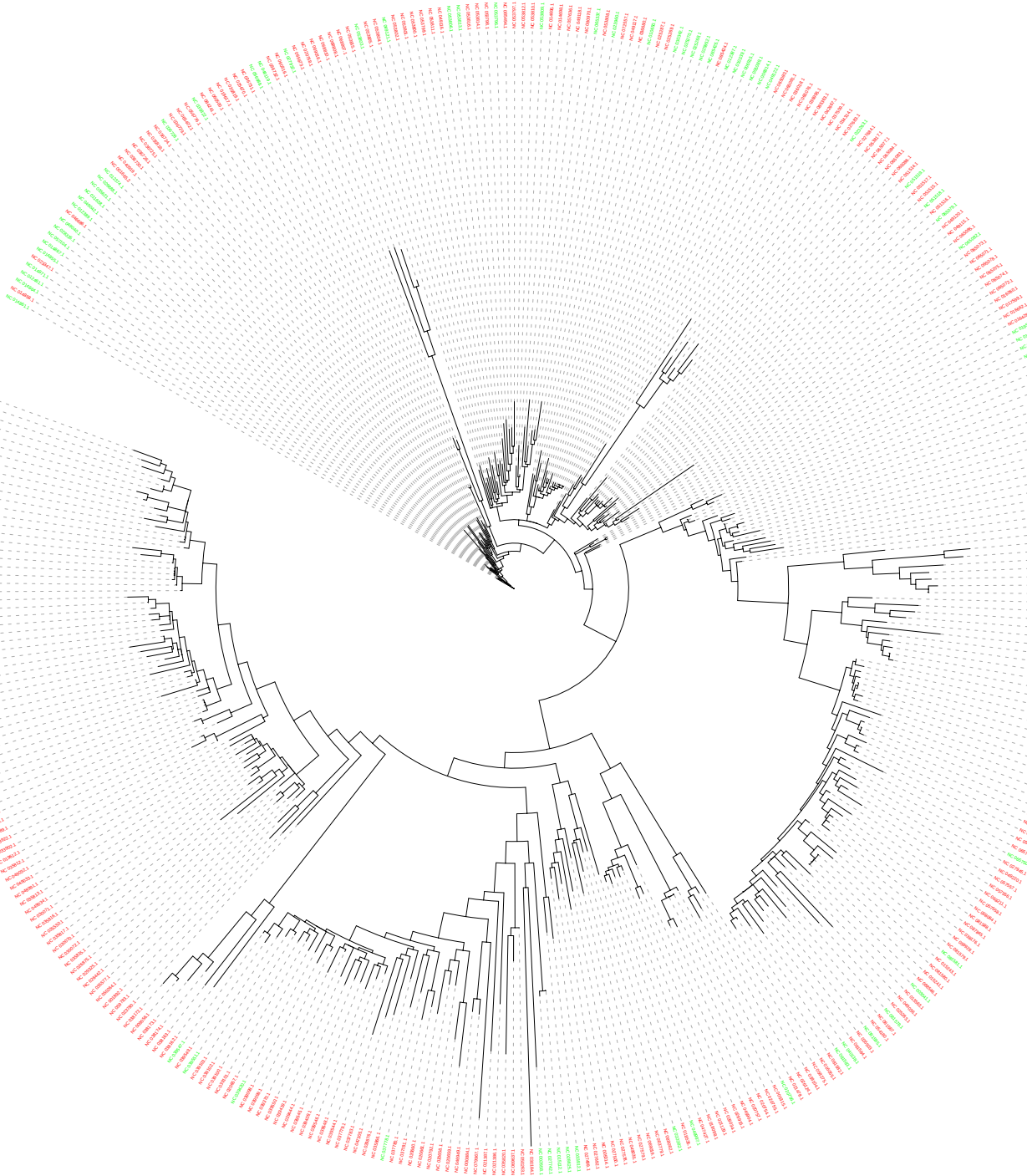

Supplement: Supplementary file 1 [file genes-16-00833-s001.zip › genes-3747614-supplementary/Figure S2 Phylogenetic tree of cytochrome b encoded proteins in Rodentia.pdf]
